# Single-Cell Sequencing Unveils the Heterogeneity of Nonimmune Cells in Chronic Apical Periodontitis

**DOI:** 10.3389/fcell.2021.820274

**Published:** 2022-02-10

**Authors:** Xinwei Lin, Danlu Chi, Qingzhen Meng, Qimei Gong, Zhongchun Tong

**Affiliations:** ^1^ Department of Operative Dentistry and Endodontics, Hospital of Stomatology, Sun Yat-sen University, Guangzhou, China; ^2^ Guangdong Provincial Key Laboratory of Stomatology, Sun Yat-sen University, Guangzhou, China

**Keywords:** single-cell analysis, cell and molecular biology, cell-cell interactions, periapical periodontitis, inflammation

## Abstract

Chronic apical periodontitis (CAP) is a unique dynamic interaction between microbial invasions and host defense mechanisms, resulting in infiltration of immune cells, bone absorption, and periapical granuloma formation. To help to understand periapical tissue pathophysiology, we constituted a single-cell atlas for 26,737 high-quality cells from inflammatory periapical tissue and uncovered the complex cellular landscape. The eight types of cells, including nonimmune cells and immune cells, were identified in the periapical tissue of CAP. Considering the key roles of nonimmune cells in CAP, we emphasized osteo-like cells, basal/stromal cells, endothelial cells, and epithelial cells, and discovered their diversity and heterogeneity. The temporal profiling of genomic alterations from common CAP to typical periapical granuloma provided predictions for transcription factors and biological processes. Our study presented potential clues that the shift of inflammatory cytokines, chemokines, proteases, and growth factors initiated polymorphic cell differentiation, lymphangiogenesis, and angiogenesis during CAP.

**GRAPHICAL ABSTRACT:**
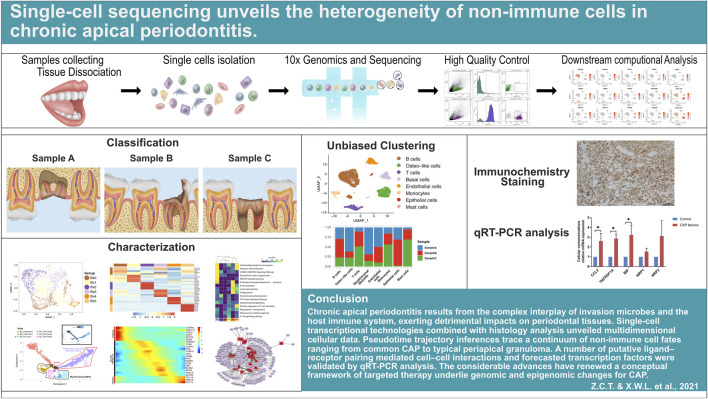
Schematic graph highlighting the overall experimental workflow for single-cell RNA sequencing and data analysis.

## 1 Introduction

Chronic apical periodontitis (CAP), caused by bacterial infection, is the most frequent tooth disease in the jaws, and manifested as periapical tissue destruction, alveolar bone resorption, and inflammatory granulation tissue formation (Chan et al., 2013). The prevalence of CAP varies from 27% to 70% and increases with advancing age (Ørstavik, 2019). The high incidence of CAP is a potential impetus for us to further study. CAP is an inflammatory disease caused by the complex interaction between various types of cellular compositions in periapical lesions, comprising immune cells and nonimmune cells. The infiltration of massive immune cells is considered to be a hallmark of pathological change in CAP. A few studies unraveled that CAP was related to the anamnestic humoral immunity mediated by lymphocytes (Hoda and Hoda, 2005; Lerner, 2006; Jain et al., 2020). The aberrant proliferation of immune cells interferes with the established cellular equilibrium in the lesion, and drives a detrimental inflammatory response to impair periapical tissue. Meanwhile, the nonimmune cells with limited intrinsic healing capacity are coordinated with lymphocytes and myeloid cells to protect against the spread of infectious agents to other locations and mediate lesion tissue remodeling.

The nonimmune cells play a key role in the outcome of pathological tissue in CAP. An imbalance of osteoblasts and osteoclasts under the immune tolerance of CAP is the main cause of progressive bone loss in the periradicular area (Braz-Silva et al., 2019). The inflammatory periapical tissue is well supplied with activated endothelial cells, and forms angiogenesis, which shields the host against microbiota composition (Al-Soudi et al., 2017). In the inflammatory environment, fibroblast, a sentinel in the periapical tissue, secretes matrix metalloproteinase to influence the extracellular matrix organization, and the heterogeneity of fibroblasts is possible to cause the formation of periapical granulomas (PGs)/radicular cysts (RCs). The PG/RC is surrounded by a fibrous capsule previously hypothesized to be formed by the remnants of epithelial cells (Malassez, ERM) and the immune cells (Omoregie et al., 2011; Subramaniam et al., 2019). Although the histological identification of inflammatory cells has highlighted the roles of immune cells, little is known about the molecular mechanism of nonimmune cells in CAP at the single-cell level.

The jaws are distinct from the other skeletal defense systems in the body due to teeth existence. The tooth defect sets up a pathway in that pathogenic bacteria in the oral cavity directly contaminate the bone marrow without any protective barriers and then develop the inflammatory periapical lesion in alveolar bone (Zohrabian and Abrahams, 2015). The recent widespread use of single-cell and high-throughput sequencing in many fields provided an opportunity to comprehensively understand cellular heterogeneity and multiple biological processes of nonimmune cells in odontogenic CAP. The tissue hyperplasia cells and transcriptome genes from common chronic periapical periodontitis to a significantly periapical granuloma were perceived by exploiting single-cell transcriptomics. Polymorphic cells were detected in pathogenesis and healing processes of CAP, reflecting the dynamic interaction between the inflammatory response and tissue repair/regeneration. Furthermore, lymphangiogenesis and angiogenesis were predicted by the abnormal activation of endothelial cells and regulatory program, which implied immunological feedback from the host system in CAP.

## 2 Materials and Methods

### 2.1 Patient Samples

**FIGURE 1:**
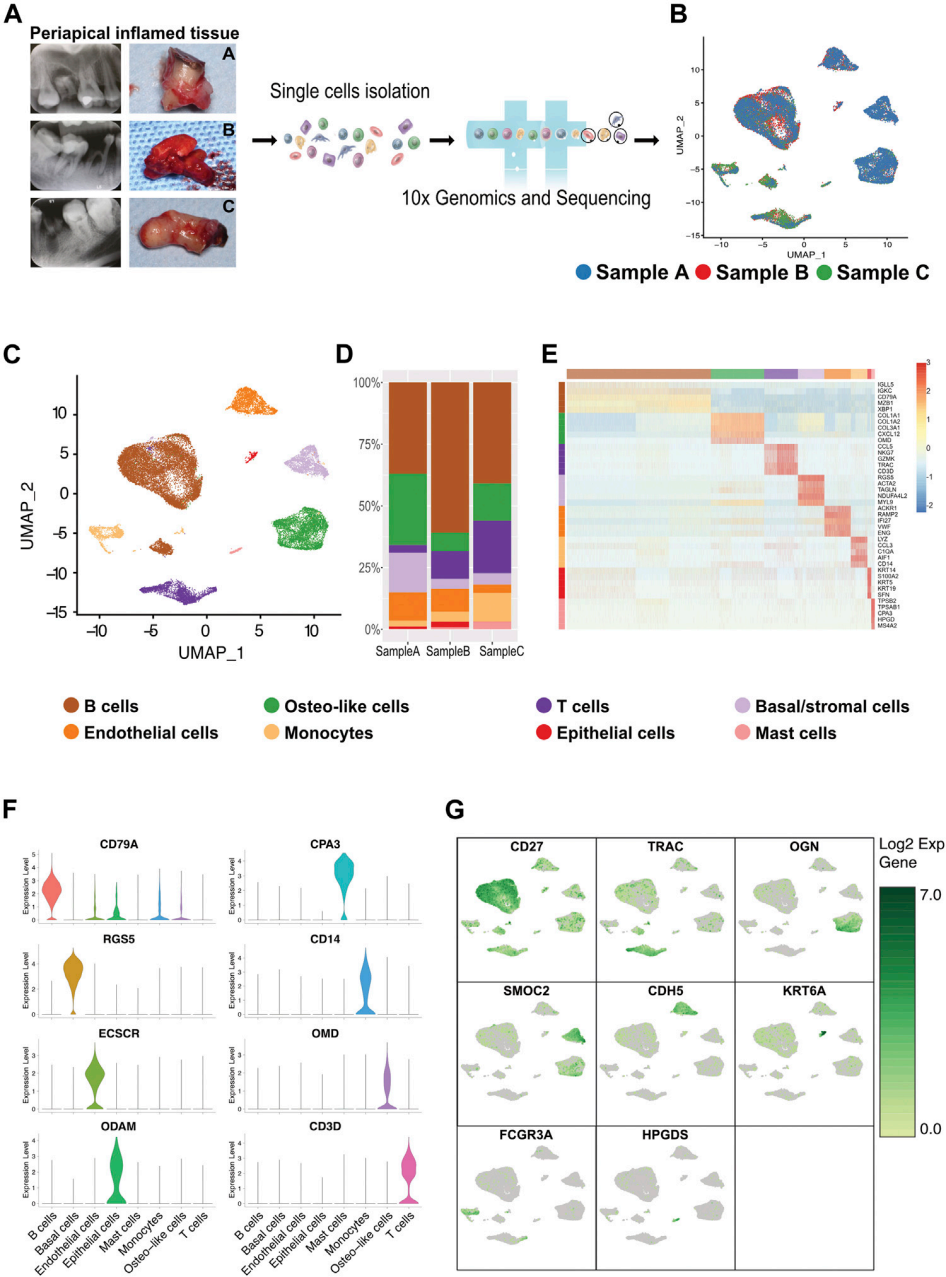
Heterogeneity of cells of human inflammatory periapical tissue in three patient individuals by single-cell RNA-seq analysis. **(A)** The intraoral and x-ray examination for patients at three different states of CAP (*n* = 3; Samples A and B were from common CAP and Sample C was from typical periapical granulomas). **(B)** The single-cell RNA-seq profiling of inflamed human periapical tissue reveals the cell proportion and gene clusters in three different states in a uniform manifold approximation and projection (UMAP) plot combined from 3 patients. Moderate blue (3377B3), bright red (EA382C), and dark moderate lime green (479F32) represent Samples A, B, and C, respectively. **(C)** The UMAP dimensionality reduction plot, in combination with scientific color data, is clustered into eight cell types. The cell cluster phenotype is noted in the color key legend and labels. Each point depicts a single cell, colored according to the cell type. **(D)** The bar plot of the major cell types and cell proportions (center). **(E)** The heatmap showing the relative expression of the top 5 [by average log (fold change)] genes in each cell type (right). The heatmap of the top 20 [by average log (fold change)] marker genes from each cluster and cell type assignment of each cluster is shown in Supplementary Figure S5. The canonical markers for each cell type are color-coded and shown below. **(F)** The violin plot of the marker genes for each major cell cluster. **(G)** The UMAP depicting significant (canonical gene markers) gene expression of single-cell clusters in combined specimens (*n* = 3).

Based on alveolar bone destruction shown *via* apical x-ray examinations, three residual molars with CAP were extracted due to incurability. Sample A (female, 26 years) and Sample B (male, 44 years) were from common CAP (the smaller pathological tissue), and Sample C (female, 27 years) was from a typical periapical granuloma (the bigger pathological tissue) (Figure 1A). Another three samples of periapical tissue were collected, and immunohistochemistry staining was performed to validate the cellular compositions in CAP (seen in Section 2.10). Furthermore, we collected three other samples of periapical tissue to validate the expression of the key genes in cell–cell interactions (CCIs) and transcription factors (TFs) by quantitative real-time polymerase chain reaction. The normal periapical tissue was scraped from the surface of roots of three premolar teeth that were extracted due to orthodontic treatment, and referred to as the controls (seen in Section 2.9). The patients were excluded the autoimmune diseases, and did not take any antibiotics in the recent 3 months. Inflammatory periapical tissues were collected for research approved by the Medical Ethics Committee of Hospital of Stomatology, Sun Yat-sen University (Seal) (KQEC-2020-67-01). Our study had no influence on the fate of the extracted teeth at any point, and we complied with all relevant ethical regulations.

### 2.2 Tissue Dissociation and Preparation of Single-Cell Suspensions

Once the inflammatory periapical tissue of the teeth was carefully scraped off, the collected samples were immediately placed in an ice-cold preservation solution and then transported to the laboratory to maintain viability. The following progress was performed as outlined by the 10x Genomics Single Cell 3′ v2 Reagent Kit user guide (10 X Genomics, 2019a). After being mechanically dissected into 1- to 2-mm small pieces, the tissue fragments were enzymatically dissociated in 10 ml of solution containing 1 mg/ml collagenase type I (Gibco, United States; #17100-017), 2 mg/ml dispase II (Sigma-Aldrich; #D4693-1G), 0.5 mg/ml elastase (Solarbio; #E8210), and 1 unit/ml DNase I (NEB; #M0303S) in PBS with 1% FBS for 30 min by gentle stirring 6 times in a 37°C water bath. Subsequently, the disaggregated tissue components were filtered through a 70-µm cell strainer and lysed with 1X RBC lysis buffer to remove red blood cells. The cell pellets were resuspended in PBS (Life Technologies) with 0.4% BSA (Sigma) before determining the sample quality. Cell viabilities were determined *via* Trypan blue (Thermo Fisher, United States) staining. The cell yields were estimated using an automatic cell counter (FL-CD, Countstar, China), and CD45 monoclonal antibodies were detected by flow cytometry (Supplementary Figure S1). Single-cell suspensions were sorted in MoFlo Astrios (Beckman Coulter, United States), ensuring >80% viability, and adjusted to a concentration of approximately 1 × 10^6^ cells/ml.

### 2.3 10x Single-Cell Processing and cDNA Library Construction

Using the manufacturer’s protocol (10x Single Cell 3′ Reagent Kits v2 manual, United States) (10 X Genomics, 2016), the prepared cell suspensions were loaded onto barcoded scRNA-seq. In brief, the generation of gel beads in emulsions (GEMs), barcoding, GEM-RT clean-up, complementary DNA amplification, and library construction were performed. The quality and molarity of each library were ensured based on library size as measured using a bioanalyzer (Agilent Technologies, United States) and qPCR amplification data. The libraries were subjected to high-throughput sequencing on an Illumina NovaSeq 6000 PE150 platform (Southard-Smith et al., 2020) as 150-bp paired-end reads at one full lane per sample.

### 2.4 Computation and Quality Control

Raw sequencing reads were demultiplexed by bcl2fastq software to convert them into 150-bp paired-end reads and stored in the FASTQ format. The FASTQ files were aligned to the GRCh38 human reference genome through the Cell Ranger Single-Cell Software Suite (version 3.1.0) (10 X Genomics, 2019b) to develop digital gene expression matrices. Subsequently, unique molecular identifier (UMI) counting was obtained by uniquely mapping the exonic reads to the transcriptome. In addition to discarding the genes expressed in fewer than three cells, cells with fewer than 200 detected genes, greater than 30,000 UMIs, or more than 10% of reads from mitochondrial genes were also treated as low-quality cells that needed to be removed. To normalize and scale the single-cell gene expression data, we imported the filtered single cells and the UMI count matrices into the Seurat (v3.1.5) R package (v3.5.2, https://www.R-project.org/) (Satija et al., 2015; Butler et al., 2018). By the Seurat “FindVariableGenes” function, we determined the highly variable genes (HGVs) across the single cells in each sample (Luecken et al., 2021). Following principal component analysis (PCA), the first 20 principal components were selected for cell clustering and dimension reduction visualized by t-SNE and UMAP plots at a resolution of 0.5 (Supplementary Figure S3). We utilized K-means clustering to determine that the resulting subpopulations were similar to those from spectral clustering, as judged by differential expression analysis, reflecting the steadiness of our approach.

### 2.5 Differential Gene Expression Analysis

Per the above clustering analysis, the marker genes for each cluster were detected by using differential expression analysis between cells. We applied the edgeR package (Robinson et al., 2009) to determine differentially expressed genes between different groups with |Log2-fold change | > 0.5 and *p*-value < 0.05 as thresholds. Hierarchical clustering was performed on the average RNA cluster, and genes were aggregated from the literature and visualized using Complex Heatmap (v.1.20.0) (Gu and Hübschmann, 2021). In clusterProfiler, we performed enrichment functional annotation of the grouped genes with similar trends in expression based on GO distribution at a specific level (Boyle et al., 2004).

### 2.6 Pseudotime Ordering and Lineage Trajectories

The single-cell pseudotime trajectories were generated with the Monocle2 R package (v2.10.1) (Reid and Wernisch, 2016; Trapnell and Cacchiarelli, 2014) by computing and ordering the gene expression changes of the collected cells. The gene-cell matrix at the scale of the raw UMI counts derived from the Seurat processed data was used. To validate the results from Monocle 2, the slingshot algorithm, a popular trajectory analysis tool that fits the bifurcation trajectory, was reanalyzed to determine the cellular trajectory of the four types of nonimmune cell osteo-like cells, basal/stromal cells, endothelial cells, and epithelial cells. For temporal profiling of the dynamic changes in branch-dependent gene expression levels during CAP (i.e., along specific lineages), we constructed a special type of heatmap in which genes with similar lineage-dependent expression patterns were clustered together (Figures 4F, 5F, 6F, 7F).

### 2.7 Pathway Enrichment Analysis

With our submitted multiple gene lists, we exploited Gene Ontology (GO) and KEGG pathway enrichment analyses to further obtain in-depth information on the functional and mechanistic insights of a cell cluster by Metascape (http://metascape.org/) (Zhou et al., 2019). After mapping to the NCBI Human Homologene database, all statistically enriched terms, including the GO/KEGG terms, canonical pathways, and hallmark gene sets, were hierarchically clustered into a tree through Metascape, and Kappa-statistical similarities were utilized to identify the involved biological pathways. Heatmaps were employed to visualize a few suitable biological pathways for each cell cluster (Figures 4D, 5D, 6D, 7D).

### 2.8 Transcription Factors Predicted in TRRUST

We predicted the cell type-specific TFs of nonimmune cells in CAP lesions by applying different gene expression levels in each cell state in TRRUST. Inflammatory factors in periapical tissue might activate different TFs to coordinate abnormal gene expression and regulate cell function in different states. The inhibition or activation domain is the core of the regulatory mechanism of TFs as well as their correlated gene expression. Their specific combination might downregulate or upregulate the target gene expression, leading to different cellular reactions. As these TFs are activated in different stimuli, specific cell types might coordinate cell type-specific functions and express aberrant genes by distinct signaling pathways (Han et al., 2018).

### 2.9 The Validation by Quantitative Real-Time Polymerase Chain Reaction

We extracted and purified total RNA from three periapical inflammatory tissue of the freshly extracted teeth (Lesion group) and the scraped sample of normal teeth roots (The controls) using the RNeasy Mini Kit (Qiagen, Antwerp, Belgium) according to the manufacturer’s protocol. RNA concentration was assessed using a NanoDrop ND 1000 Spectrophotometer (NanoDrop Technologies, Wilmington, DE, United States). cDNA was synthesized using a reverse transcriptase kit (TaKaRa, Japan), followed by qRT–PCR analysis using the Hieff™ qPCR SYBR® Green Master Mix (Yeasen). Quantitative PCR reactions were performed with a QuantStudio5 Cycler (Applied Biosystems, United States), together with human β-actin as the housekeeping gene. The primers were designed on the website of Primer Bank (https://pga.mgh.harvard.edu/primerbank/). The following sequences were used (Table 1):

**TABLE 1:** Table of primer sequences for RT-qPCR.

Gene	Primers	Length	Annealing temperature
CCL2	Forward: 5′-AAA​CTG​AAG​CTC​GCA​CTC​TCG-3′	115 bp	60.93
	Reverse: 5′-TTG​ATT​GCA​TCT​GGC​TGA​GCG-3′		61.61
TNFRSF1A	Forward: 5′-GAG​AAT​GTT​AAG​GGC​ACT​GAG-3′	150 bp	56.33
	Reverse: 5′-CCC​ACA​AAC​AAT​GGA​GTA​GA-3′		54.96
MIF	Forward: 5′-CAG​TGG​TGT​CCG​AGA​AGT​CAG-3′	440 bp	60.34
	Reverse: 5′-TAG​GCG​AAG​GTG​GAG​TTG​TT-3′		58.95
NRP1	Forward: 5′-ATC​ACG​TGC​AGC​TCA​AGT​GG-3′	168 bp	60.95
	Reverse: 5′-TCA​TGC​AGT​GGG​CAG​AGT​TC-3′		60.32
NRP2	Forward: 5′-GTG​ACT​GGA​CAG​ACT​CCA​AG-3′	543 bp	57.55
	Reverse: 5′-CGA​ACA​CAA​TCT​GGT​ACT​CC-3′		55.89
RFX5	Forward: 5′-GAT​GAG​CCT​GAT​GCT​AAG​AGC-3′	134 bp	58.58
	Reverse: 5′-CCC​TCT​ACT​TTG​TTC​TGC​ACG-3′		58.93
NF-κB	Forward: 5′-ACA​CCG​TGT​AAA​CCA​AAG​CC-3′	209 bp	58.97
	Reverse: 5′-CAG​CCA​GTG​TTG​TGA​TTG​CT-3′		59.04
STAT3	Forward: 5′-CTG​CCC​CAT​ACC​TGA​AGA​CC-3′	200 bp	59.82
	Reverse: 5′-TCC​TCA​CAT​GGG​GGA​GGT​AG-3′		60.03
KLF4	Forward: 5′-GTC​CCG​GGG​ATT​TGT​AGC​TC-3′	546 bp	60.18
	Reverse: 5′-TGT​AGT​GCT​TTC​TGG​CTG​GG-3′		59.96
SP1	Forward: 5′-TGG​CAG​CAG​TAC​CAA​TGG​C-3′	126 bp	60.68
	Reverse: 5′-CCA​GGT​AGT​CCT​GTC​AGA​ACT​T-3′		59.10
HIF1A	Forward: 5′-CCA​CAG​GAC​AGT​ACA​GGA​TG-3′	150 bp	57.32
	Reverse: 5′-TCA​AGT​CGT​GCT​GAA​TAA​TAC​C-3′		56.27
TWIST2	Forward: 5′-GCA​AGA​TCC​AGA​CGC​TCA​AGC​T-3′	138 bp	63.18
	Reverse: 5′-ACA​CGG​AGA​AGG​CGT​AGC​TGA​G-3′		64.27
PPARG	Forward: 5′-AGG​AGC​AGA​GCA​AAG​AGG-3′	169 bp	56.21
	Reverse: 5′-TAA​ATG​ATC​TCG​TGG​ACT​CCA​TAT​T-3′		57.7

### 2.10 Histology Analysis

Immunohistochemistry staining was performed to characterize the main cell types according to the following standard procedures. Briefly, human periapical hyperplasia tissues were rapidly harvested and fixed in 4% paraformaldehyde. After being mounted, the sections were dried in a 65°C oven (HGZF-101-1) for 2 h in preparation for deparaffinization. Slides were dehydrated with sequential washes in ethanol (100%, 100%, 95%, and 80%) and purified water for 5 min each. The slides were cut into sections with a thickness of 4 μm, treated in a 0.1 M sodium citrate buffer, and then heated for antigen retrieval. After cooling, the antigen retrieval buffers were discarded, and the sections were placed into a wet box. Endogenous peroxidase was blocked by incubation with 3% (vol/vol) H_2_O_2_ in distilled water for 30 min. The sections were rinsed in PBS at room temperature 3 times for 5 min each time. Next, the sections were incubated overnight with primary antibodies against CD3 (1:200 Proteintech; #17617-1-AP), CD19 (1:200 Bioss; #bs-0079), CD14 (1:50 Proteintech; #17000-1-AP), CD117 (1:200 Proteintech; #18696-1-AP), CTSK (1:200 BOSTER; #PB9856), BCAM (1:200 BOSTER; #A03148-1), VWF (1:200 ABCAM; #A03145-1), and ODAM (1:200 Proteintech; #16509-1-AP) at 4°C. The secondary horseradish peroxidase-conjugated goat anti-rabbit antibody (1:100; #ZB-2301) for immunohistochemistry was added at 37°C for 30 min. The sections were visualized with diaminobenzidine (DAB) staining and hematoxylin counterstaining. Images were taken by an Olympus BX43 microscope (Center Valley, PA).

### 2.11 Statistics

All statistical analyses were performed using GraphPad Prism Software (Prism 8.0) and R software (version 4.1.0). Statistical significance was assessed by the unpaired two-tailed Student’s *t*-test. *p* values of ≤0.05 were considered statistically significant. For each experiment, the tissue samples from a single patient were processed individually. Single-cell suspensions for each sample were used in scRNA-seq (10x Genomics) in an independent Chromium chip.

## 3 Results

### 3.1 scRNA-Seq Mapping of Cellular Heterogeneity in CAP Tissue

**FIGURE 2:**
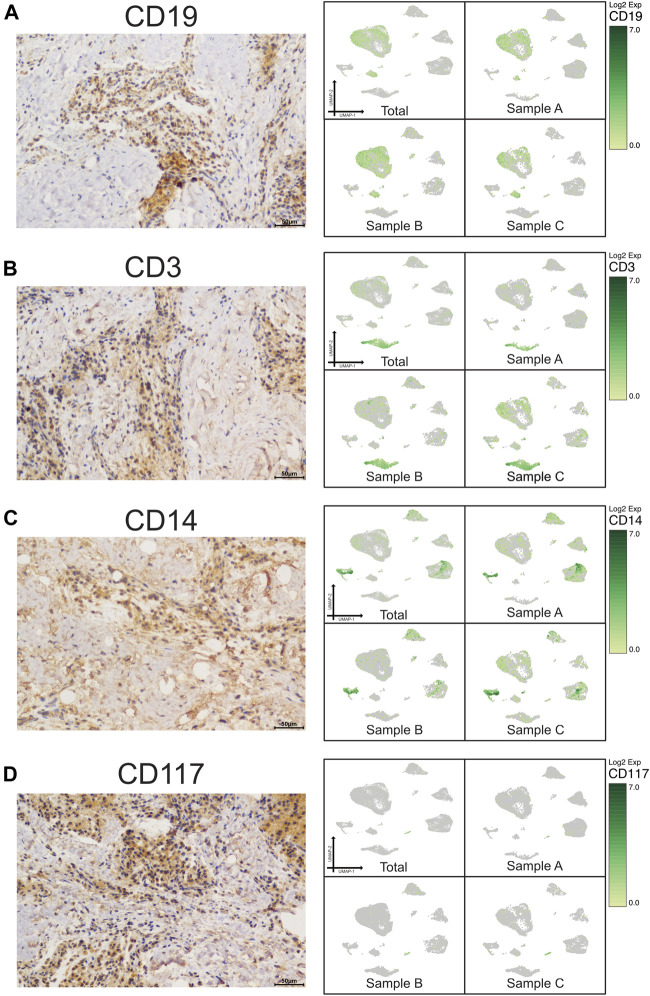
Validation of UMAP data with immunochemistry analysis of immune cells in CAP (antibody-positive). Immunochemistry staining presented the location of CD19 for B cells **(A)**, CD3 for T cells **(B)**, CD14 for monocytes **(C)**, and CD117 for mast cells **(D)** in a subset of CAP lesions (left micrograph). Images were taken at ×400 magnification. Scale bars, 50 μm. The relevant transcriptome data visualized in a UMAP plot from the three samples (right pictures).

**FIGURE 3:**
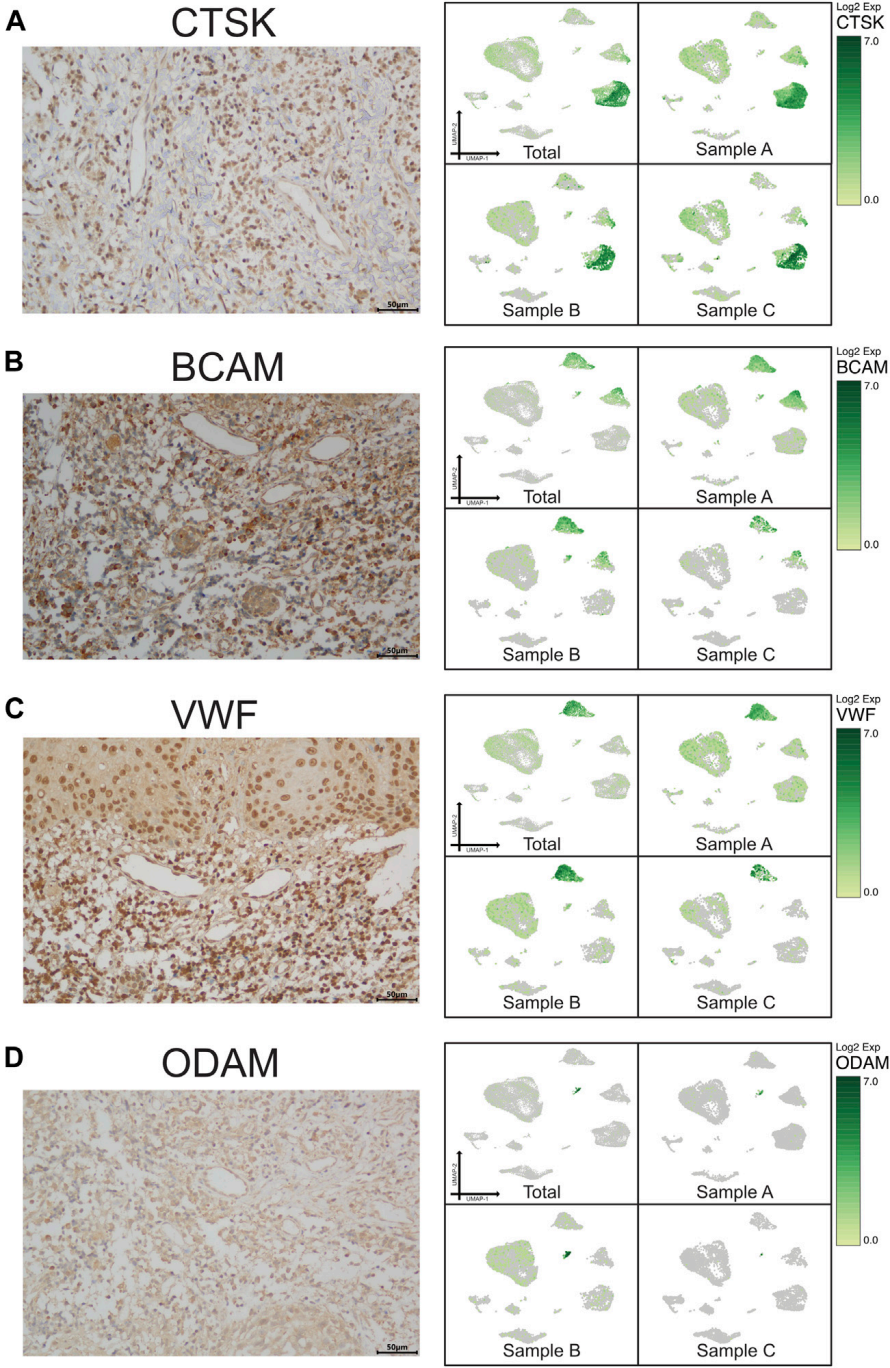
Validation of UMAP data with immunochemistry analysis of nonimmune cells in CAP (antibody-positive). Immunochemistry staining showed the location of CTSK for osteo-like (osteoclast) cells **(A)**, BCAM for basal/stromal cells **(B)**, VWF for endothelial cells **(C)**, and ODAM for odontogenic epithelial cells **(D)** in a subset of CAP lesions (left micrograph). Images were taken at ×400 magnification. Scale bars, 50 μm. The relevant transcriptome data visualized in a UMAP plot from the three samples (right pictures).

A scRNA-seq analysis was implemented to explore the cellular composition and characterizations of local periapical lesions (Figure 1A). We obtained a total of 26,737 cells from two common CAPs (Samples A and B) and a typical periapical granuloma (Sample C), including 9,868 cells from Sample A, 9,857 cells from Sample B, and 7,012 cells from Sample C. The 10X data were analyzed by the Seurat R packages (Huang et al., 2021; Zhou and Jin, 2020) for quality control, normalization, and batch effect correction, and DoubletFinder was used to perform doublet removal (Supplementary Figure S2) (McGinnis et al., 2019). Subsequently, principal component analyses were executed for dimensionality reduction (Supplementary Figure S3) (Quinn and Keough, 2012). We further utilized unsupervised graph clustering to partition the cells into clusters and visualized the clusters *via* T-distributed stochastic neighbor embedding (t-SNE) (Supplementary Figure S4) and uniform manifold approximation and projection (UMAP) (Figures 1B,C). The “Cell Ranger” tool was used to define the cellular identity of each cluster (Patel, 2018). We selected the marker genes from the published literature to identify the four types of immune cells, such as CD79A and CD27 for B cells (Mason et al., 1995; Agematsu et al., 2000), TRAC, NKG7, and CD3D for T cells (Ng et al., 2020; Stadtmauer et al., 2020; Wang Z. et al., 2021), CD14 and CD68 for monocytes (Wright et al., 1990; Iqbal et al., 2014), and TPSB2, TPSAB1 and CPA3 for mast cells (Alnaeeli et al., 2006; Merluzzi et al., 2015; Finlin et al., 2017; Wilcock et al., 2019). Moreover, we recognized the four types of nonimmune cells by multiple canonical marker genes, for example, OMD, COL1A1, and TNFRSF11B for osteocytes (Lee et al., 2006; Baniwal et al., 2012; Prideaux et al., 2014; Kartha et al., 2016); ODAM (odontogenesis), KRT19, and KRT14 for epithelial cells (Nozato et al., 2013; Cunha et al., 2017; Springer et al., 2019); BCAM, RGS5, and TAGLN for basal/stromal cells (Mitchell et al., 2008; Yu et al., 2013; Elsafadi et al., 2016); and ACKR1, VWF, and CDH5 for endothelial cells (Sumpio et al., 2002; Diacovo, 2007; Sauteur et al., 2014) (Figures 1E–G and Supplementary Figure S5). In total, eight cell types, B cells, T cells, monocytes, mast cells, osteo-like cells, basal/stromal cells, endothelial cells, and epithelial cells, were detected in the three samples according to the mentioned markers to gain an understanding of the cellular heterogeneity of periapical tissue in CAP. Immunohistochemistry staining depicted the main cell types in CAP tissues diagnosed by the same clinician. In immune cells, CD3, CD19, CD14, and CD117 were respectively marked for T cells, B cells, monocytes, and mast cells (Figure 2). In nonimmune cells, CTSK, BCAM, VWF, and ODAM were respectively coded for osteo-like cells, basal/stromal cells, endothelial cells, and epithelial cells (Figure 3). These findings presented the pleiotropic characterizations of nonimmune cells and raised our curiosity to acquaint the full potential of nonimmune cells in CAP.

### 3.2 Bone Resorption Based on the Participation of Lymphocytes, Myeloid Cells, and Osteoblastic and Osteoclastic Cells in CAP

**FIGURE 4:**
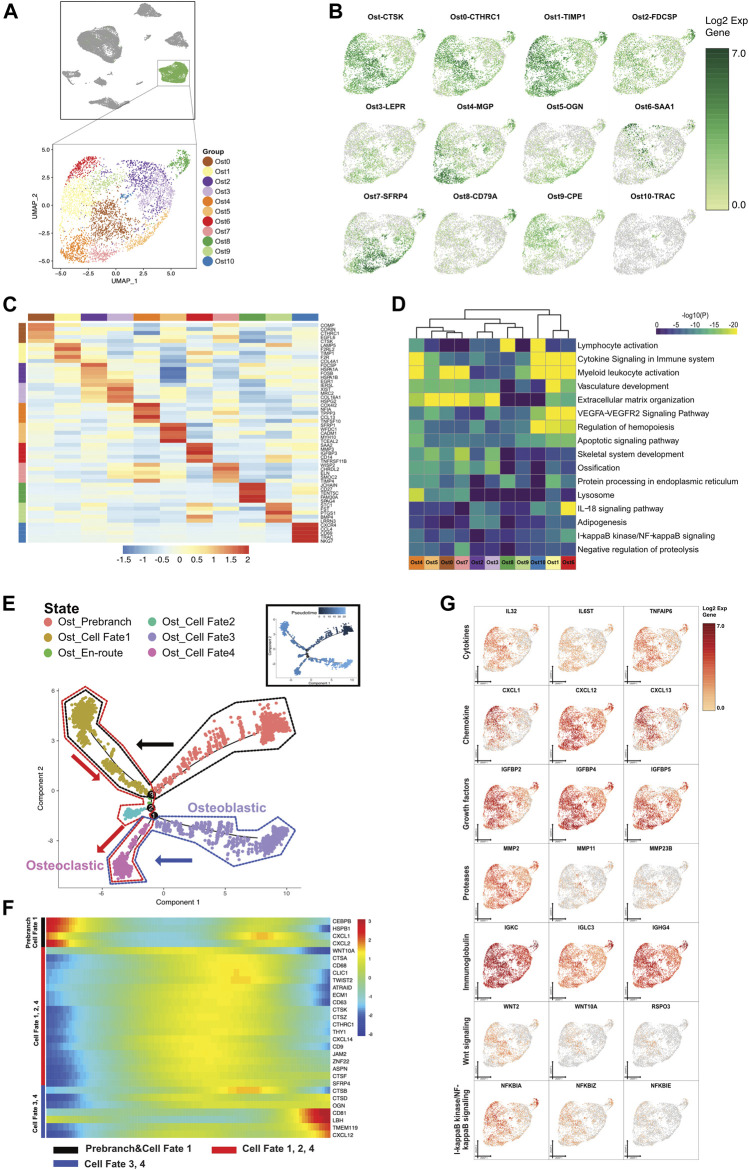
A single-cell atlas for characterizing osteo-like cells. **(A)** The UMAP plot presents the distribution of the osteo-like cell cluster (green, *n* = 4,631) in the atlas (top), and the enlarged UMAP plot (bottom) shows the eleven subclusters of the osteo-like cell populations (color coded). **(B)** The feature plots (UMAP) displaying the main signatures for each subcluster of osteo-like cells. CTSK stands for osteoclast marker. **(C)** The heatmap showing the top 5 key differentially expressed genes [by average log (fold change)] from each osteo-like cell subcluster. **(D)** The heatmap showing the corresponding enriched pathways of the eleven osteo-like subtypes [by average log (fold change)]. **(E)** The differentiation pseudotime trajectory analysis. Prebranch cells are soft red (E87D72) in color and are classified into the different numbered cell fates according to the different branches. Moderate lime green (53B74C) represents the Ost_en route, illustrating condition cells before branching. Osteo-like cells are displayed in moderate yellow (BD9C33) for cell fate 1, soft magenta (E96BD2) for cell fate 2, purple (A18BF8) for cell fate 3, and pink (E46EDD) for cell fate 4. Every dot indicates one individual cell colored according to its cluster label. The inlet plot shows each cell with a pseudotime score from dark blue to light blue, which reveals the progression from common periapical inflammatory to typical granuloma state. **(F)** The DEGs in rows along the pseudotime are hierarchically clustered into three subclusters on behalf of each of the osteo-like subpopulation cell fates. **(G)** Relative responses or signaling pathways are associated with alterations in inflammatory cytokines, chemokines, and growth factors and are visualized in a UMAP plot. Dynamical changes are shown in the Wnt and NF-κB signaling pathways.

In nonimmune cell clusters, a cluster of osteo-like cells (Ost) exerts paradoxical roles with osteogenic and osteoclastic biological characteristics. To further understand the cellular constitutions, the UMAP algorithm was portrayed to identify and segregate the cluster of Ost into 11 cell subclusters by marker genes according to their intrinsic nature or their features under inflammatory responses (Figures 4A–C). In the differentiation of Ost, osteoclasts played a primary role in bone resorption. Osteoclasts were differentiated from myeloid progenitor cells that were bipotently differentiated into granulocytes and macrophages in hematopoiesis, and gave rise to osteoclast development under the inflammatory attacks. Furthermore, immature dendritic cells, under the stimulation by M-CSF and RANKL, also act as a precursor cell of osteoclasts, which are involved in bone absorption by immunoregulation (Rivollier et al., 2004). The distinctive transformation from osteoclast precursors might influence the osteolysis of alveolar bone in CAP, prompting our search for their gene signature and biological information.

#### 3.2.1 Diversity of Osteo-like Cells (Ost)

Based on the GO significant enrichment analysis in the heatmap (Figure 4D), the differential gene expression in osteo-like cells was related to bone matrix organization and degradation, cellular apoptosis, oxidative phosphorylation, and immune cell activation. We also uncovered genes encoding vasculature development and adipogenesis in osteo-like subclusters. Of interest, 6 Ost cell subclusters (Ost0, Ost1, Ost5, Ost7, Ost8, and Ost10) expressed various relative osteoclastic cell markers, hinting that osteoclast progenitors might be differentiated from distinct origins and have multipotential in an inflammatory environment. The osteoblastic cell markers were found in the other 5 Ost cell subclusters (Ost2, Ost3, Ost4, Ost6, and Ost9). The diversity of Ost suggested that bone resorption and bone remodeling occurred simultaneously in CAP (Figures 4B–D).

#### 3.2.2 Trajectory of Ost Proceeded in CAP Lesions

To explore the osteo-like cells under inflammation, we further dissected the gene patterns involved in the Ost cell state transition using the Monocle two algorithm. The predicted osteo-like cell states were shown by the expression of function genes (Figures 4E–G and Supplementary Figure S6A). We forecasted that the cells in the Ost prebranch possessed immunotolerance according to the existence of immunoglobulins and chemokine-mediated signaling pathways. With the presence of six interleukins, four tumor necrosis factors, three growth factors, MMP11, hematopoiesis markers, and three osteoclast markers, cells in cell fate 1 engaged in osteoclast differentiation from hematopoietic stem cells. The cells in cell fate 2 were annotated redifferentiating cells under neutrophil activation involved in the immune response. The cells in cell fate 3 were the aberrant proliferating osteoblasts induced by inflammatory cytokines, chemokines, growth factors, and proteases. In view of the variations in growth factors, inflammatory cytokines, and proteases, as well as the expression of mononuclear cell markers (CD63 and CD9) and osteoclast markers (CTSK and TWIST2), the cells congregated in cell fate 4 might be the osteoclast progenitor cells that migrate near the bone and regulate bone absorption and new bone formation.

### 3.3 Fibroblast and Myofibroblast Formation Dedicated to Basal/Stromal Cells

Periapical fibroblastic cells, derived from mesenchymal cells, are natural barrier protections. Fibroblastic cells synergize with the immune system to maintain tissue homeostasis and reflect the pathological conditions of the periapical tissue (Liao et al., 2011). Under the longstanding inflammation, the fibroblasts interact with immune cells by various growth factors, chemokines, matrix metalloproteinases, and proinflammatory cytokines, and transform into proliferating and migrating states (Darby and Hewitson, 2007; Turner et al., 2018). Myofibroblasts (MFs) originate mostly from fibroblasts, but are also produced from mesenchymal cells. MFs provide insights into their components including smooth muscles and fibroblasts to varying degrees from the cell morphological features (Michalik et al., 2018). MFs, a type of large and elongated cells, both facilitate collagen secretion as fibroblasts and have short-lived reversible contractility as smooth muscle cells (Jendzjowsky and Kelly, 2019). Though we have understood the key roles of fibroblasts as proliferating cell components in CAP, several fundamental questions about MFs and inflammation-associated fibroblasts (IAFs) in this disease remain unclear. Notably, Bs detected in CAP were related to multipotential stem cells from the expression of marker genes, which indicated that progenitor cells were capable of differentiating into MFs and IAFs.

#### 3.3.1 Diversity of Basal/Stromal Cells

**FIGURE 5:**
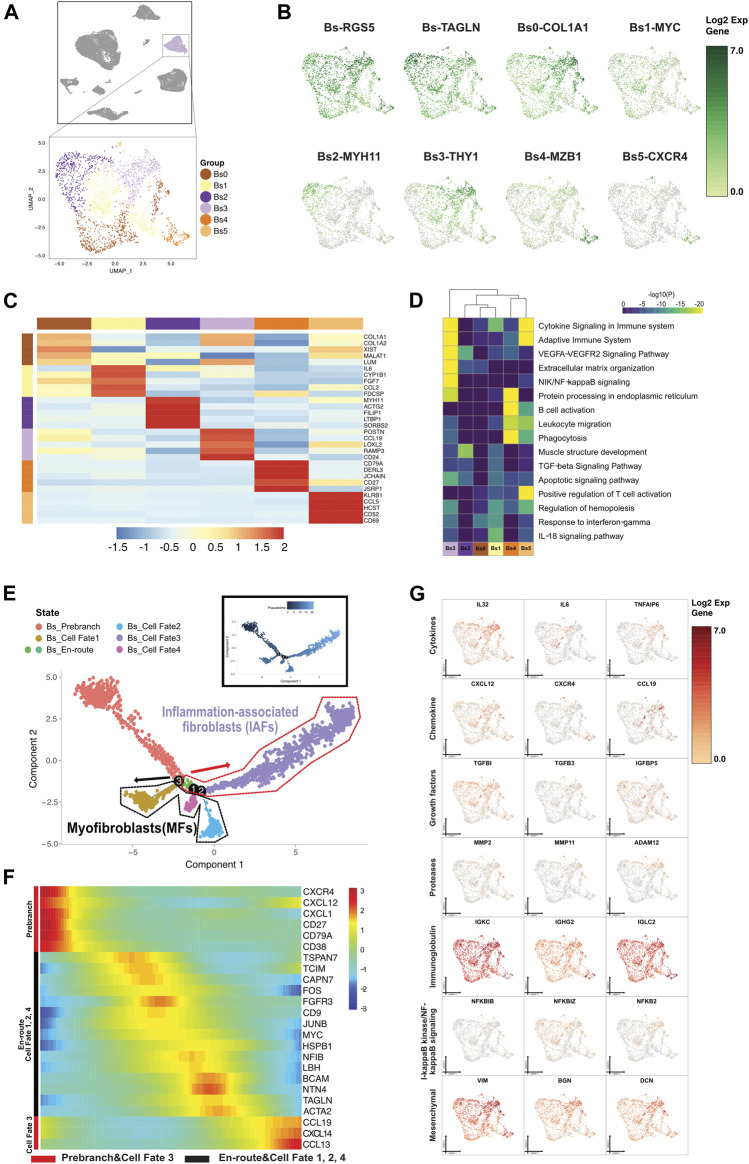
A single-cell atlas for characterizing basal/stromal cells. **(A)** The UMAP plot presents the distribution of the basal/stromal cell cluster (light purple, *n* = 2,296) in the atlas (top), and the enlarged UMAP plot (bottom) shows the six subclusters of Bs cell populations (color coded). **(B)** The feature plots (UMAP) displaying the main signatures for the subclusters of basal/stromal cells. RGS5 and TAGLN are fibroblast cell markers. **(C)** The heatmap showing the top 5 key differentially expressed genes [by average log (fold change)] from the basal/stromal cell subcluster. **(D)** The heatmap showing the corresponding enriched pathways of the six basal subtypes [by average log (fold change)]. **(E)** The differentiation pseudotime trajectory analysis. Prebranch cells are soft red (E87D72) in color and are classified into the different numbered cell fates according to different branches. Moderate lime green (53B74C) and soft magenta (E96BD2) represent the Bs_en-route, illustrating the cell condition before branching. Bs cells are moderate yellow (BD9C33) for cell fate 1, soft blue (51B4E6) for cell fate 2, purple (A18BF8) for cell fate 3, and pink (E46EDD) for cell fate 4. Each dot represents a single cell colored according to its cluster label. **(F)** The DEGs in rows along the pseudotime are hierarchically clustered into two subclusters, representing each basal subpopulation’s cell fate. The response related to variations in cytokines and chemokines occurs in the buildup of inflammatory-associated fibroblasts (IAFs). Alteration of growth factors accompanied by muscle cell genes optimize the development of myoblasts and myofibroblasts. **(G)** Relative response- or signaling pathway-associated changes in gene expression are visualized in a UMAP plot.

In this study, basal/stromal cells (Bs) were divided into 6 subpopulations by marker genes (Figures 5A–C). The classification of all Bs subclusters allowed for subtle features and functions, despite the marker gene expression for basal fibroblast or smooth muscle. The expression of genes correlated with extracellular matrix/structure organization (Li et al., 2021) was partly found in Bs0, Bs2, and Bs3, indicating that the three subclusters corresponded to fibroblast development. MFs were also considered to be transited from smooth muscle cells according to phenotypic characteristics and structural changes in the muscularis (Sferra et al., 2019). Cells in Bs2 coexpressed smooth muscle cells and myofibroblasts, as previously suggested by their expression of MYH11, ACTG2, and DES of smooth muscle cells and TAGLN and ACTA2 of myofibroblasts. Bs1, predominantly expressed FGF7, was annotated as inflammation-associated fibroblasts (IAFs) according to the high expression of CCL2, IL6, and IRF1. Bs4 expressed B-cell-specific markers and regulated endoplasmic reticulum, whereas Bs5 were related to T-cell activation and their genes involved in cytokine signaling in the immune system.

#### 3.3.2 Lineage Bifurcation in Basal/Stromal Cells Differentiation Trajectory

We reconstituted the differentiation trajectory with a total of 2,296 Bs cells that passed quality control. The Bs states in cell fates were shown by the expression of function genes, and Bs differentiation trajectory showed lineage bifurcation (Figures 5E–G and Supplementary Figure S6B). From prebranch to cell fates 1, 2, and 4, the development and differentiation of Bs were not driven by inflammatory factors due to the absence of chemokines. We annotated the cells in cell fate 1 as early differentiating cells by the unique presence of insulin-like growth factor binding protein 5 (IGFBP5) (Kiepe et al., 2006). Both cells in cell fate 2 and cell fate 4, with the expression of IGFBP7, were defined as progressively maturing MFs. Additionally, the cells in cell fate 2, due to the expression of TNFRSF12A, five relevant growth factors (CTGF, PGF, NGF, TGFβ1I1, and FGFR1OP2), and three contractile genes (MYH11, ACTG2, and DES), were also classified as contracting MFs. Under a proinflammatory (IL10RB, IL15, IL32, IL34, IL13RA1, ILF2, IL1R1, and IL6) environment, cells from prebranch to cell fate 3 showed alterations in the chemokines (CXCL1, CXCL2, CXCL12, and CXCL14) and the expression of growth factors (IGFBP2, IGFBP4, TGFβRAP1, TGFβI, TGFβ1I1 ,and TGFβ3) and proteases (MMP2 and MMP11). We identified this unique fibroblast subset expressing interleukin 6 (IL6), motif chemokine ligand 19 (CCL19), and interferon-alpha inducible protein 27 (IFI27) in cell fate 3 and therefore labeled them as inflammation-associated fibroblasts (IAFs). We also determined some mesenchymal stromal cell markers transiently exhibited in cell fate 3, which conjectured the occurrence of mineralization (VIM, BGN, and DCN) and fibroblastic differentiation (THY1) in Bs cells.

### 3.4 Lymphangiogenesis and Angiogenesis Induced by Activation of Endothelial Cells in Periapical Inflammation

Endothelial cells (Eds), a monolayer lining the vessel wall, have been considered immunity gatekeepers to deliver defense components of cellular or humoral immunity from the circulating blood to the lesions. In a bidirectional relationship between Eds and the host immune system, Eds directly detect lesion microorganisms and transfer the signals. Meanwhile, Eds are afflicted with inflammatory factors, and result in cellular dysfunction and vasodilation (El Assar et al., 2013; Zhang et al., 2019; Sun et al., 2020). The recruitment of innate immune cells in the lesions increases vascular permeability, and the emergence of lymphatic endothelial cells modulates the inflammatory response by innate expression of NF-κB molecular pathways (Tammela and Alitalo, 2010; Jin et al., 2011). In accordance with the immune function of blood vessels, the inhibition of lymphatic sprouting might pose an imminent threat to local inflammatory tissue edema and lead to pain by compressing nerves (Käßmeyer et al., 2009). In healthy adults, Eds usually keep quiescent and do not proliferate (Ricard et al., 2021), but given this dynamic, we speculated that the endothelial cells in CAP might be transformed from bone marrow hematopoietic stem cells, myeloid cells, side populations, and tissue-residing pluripotent stem cells.

#### 3.4.1 Dividing Endothelial Cells Into Nine Prominent Cell Subgroups

**FIGURE 6:**
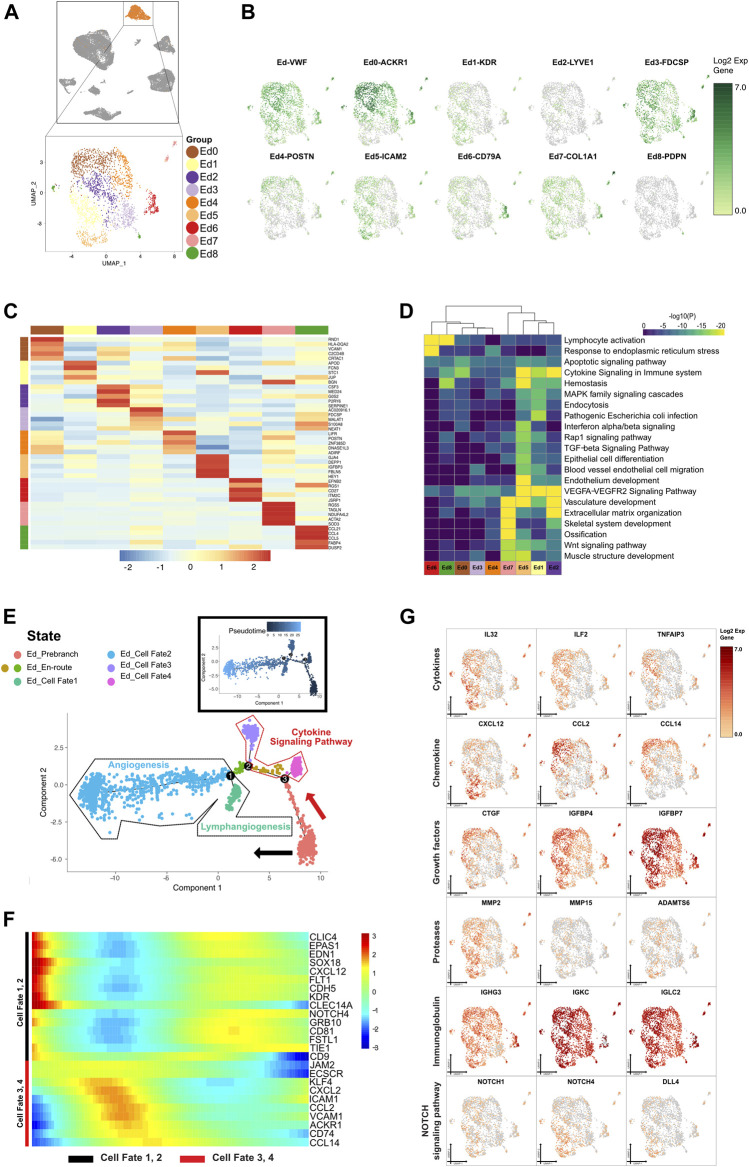
A single-cell atlas for characterizing basal/stromal cells. **(A)** The UMAP plot presents the distribution of the endothelial cluster (orange, *n* = 2,290) in the atlas (top), and the enlarged UMAP plot (bottom) shows the nine subclusters of endothelial cell populations (color coded). **(B)** The feature plots (UMAP) display the main signatures for endothelial cells. VWF is an endothelial cell marker, as previously studied. **(C)** The heatmap showing the top 5 key differentially expressed genes [by average log (fold change)] from the endothelial cell subcluster. **(D)** The heatmap showing the corresponding enriched pathways of the nine endothelial subtypes [by average log (fold change)]. **(E)** The differentiation pseudotime trajectory analysis. Prebranch cells (soft red, E87D72) are classified into the different numbered cell fates according to the different branches. The cells in the Ed_en-routes (moderate yellow, BD9C33 and moderate lime green, 53B74C) illustrate the condition the cells are in before branching. Endothelial cells in cell fate 1 (soft magenta, E96BD2), cell fate 2 (soft blue, 51B4E6), cell fate 3 (purple, A18BF8), and cell fate 4 (pink, E46EDD) are displayed. Each dot represents a single cell colored according to its cluster label. **(F)** The DEGs in rows along the pseudotime are hierarchically clustered into two subclusters, representing each endothelial subpopulation’s cell fate. Cells in cell fate 3 and 4 respond to cytokine production, while cells in cell fate 1 and 2 are enriched in vasculature development. **(G)** The relative response- or signaling pathway-associated changes in gene expression are visualized in a UMAP plot.

In this study, 8.59% of CAP cells were classified as endothelial cells. Specifically, we partitioned endothelial cells into 9 cell subclusters according to similarities among each cluster and canonical marker gene expression (Figures 6A–C). Of these, Ed0, coexpressing both myeloid and activated endothelial markers, were associated with inflammatory factors and human leukocytes, which suggested that they might be differentiated from myeloid cells in an inflammatory environment. Ed1, with expression of the cell cycling marker RGCC, hematopoietic stem cell marker CD34, and endothelial progenitor cell marker KDR, were supposed to be immature endothelial cells differentiated from hematopoietic stem cells. Ed2, characterized by lymphatic endothelial cell marker LYVE-1 and growth factor IGFBP5, were considered a population of early differentiating lymphatic endothelial cells. Ed3 were involved in blood vessel endothelial cell migration and apoptotic signaling pathway, whereas Ed4 responded to endoplasmic reticulum stress under the dynamical interaction with microorganisms. Intriguingly, Ed5 enriched in endothelial development during lymphangiogenesis. Ed6 presented proliferating endothelial cell markers and were inferred to be related to B-cell activation when responding to endoplasmic reticulum stress. Ed7, with pericyte marker (RGS5), fibroblast markers (LUM and DCN), and smooth muscle cell markers (TAGLN and ACTA2), were reckoned to be critical for vascular repair. We uncovered that Ed8 manifested T-cell activation, hemostasis, and phagocytosis by the relevant marker genes. The gene expressions in the vascular Notch pathway and VEGFA–VEGFR2 signaling pathway were ubiquitous in all Ed subsets, suggesting that the two signaling pathways might be normally activated in the endothelial cells in CAP (Figure 6D).

#### 3.4.2 Lineage Bifurcation in Endothelial Differentiation Trajectory

The sustained stimulus on periapical tissue caused the endothelial cell dysfunction of periapical inflammatory tissue. We reconstructed endothelial cell lineage relationships by pseudotemporal ordering. According to the direction and timing, the clustering of genes unveiled seven states from prebranch to the 4 different cell fates that were demarcated by CDH5 and ACKR1 expression (Figure 6E). In the presence of cell cycling markers, antimicrobial genes, tissue compartment-specific markers, and the growth factor for vasculature development, the cells in Ed_prebranch were enriched in the “organic acid metabolic process” and the “carboxylic acid metabolic process,” and thereby termed the redifferentiating cells in the inflammatory or immunoregulatory process. From prebranch to cell fate 1, the decrease of IL32, CXCL12, MMP2, and CD34 and increase of IGFBP5 and CLIC4 showed that the cells in this process might be involved in lymphangiogenesis. The expression of TF SOX18 in cell fate 1 played a critical role in triggering the differentiation of lymph vessels with the emergence of myocyte enhancer factor (MEF2C) and lymphocyte recirculation mediator (ICAM2). From prebranch to cell fate 2, Eds (KDR and VWF) contributed to angiogenesis through lymphocyte-mediated immunity and endothelial cell migration due to the alteration of gene expression including MMP15, ADAMTS4, ADAMTS6, ADAMTS9, CXCL13, IL4R, TNFAIP2, FLT1, HEG1, TIE1, IGFBP3, and IL6ST. The existence of various immunoglobulins indicated that angiogenesis was driven by an inflammatory milieu. The presence of NOTCH1 and the gradual enhancement of NOTCH4 denoted angiogenesis and lymphangiogenesis in CAP through the NOTCH signaling pathway. In the other bifurcated trajectory (cell fates 3 and 4), the activated endothelial cells (ACKR1 and VCAM1) exhibited a marked shift in their cytokine–chemokine expression profile, due to changes of expression of IL33, ILF2, TNFAIP3, CTGF, IGFBP4, IGFBP7, CCL2, CCL14, CCL23, CXCL2, and CXCL3. Myeloid cells (CD74) were intermingled in the close vicinity of the activated endothelial cells in cell fates 3 and 4, and showed their potential roles in the immune response (Figures 6E–G and Supplementary Figure S7A).

### 3.5 Identification of Epithelial Cell Subtypes and Cellular Alterations in CAP

Epithelial cells (Ep) belong to a distinctive cell type in CAP with cyst formation (tenCate, 1972; Dias et al., 2007; Lin et al., 2007, Lin et al., 2017). A previous study supplied evidence that the incidence rate of epithelial cell rests of Malassez (ERM) was approximately 52% (Nair et al., 1996). ERM in the inflamed periodontal ligament formed on the cyst was suspected to be an outcome of inflammatory cell infiltration or hyperplasia.

#### 3.5.1 Four Prominent Cell Subgroups in Epithelial Cells

**FIGURE 7:**
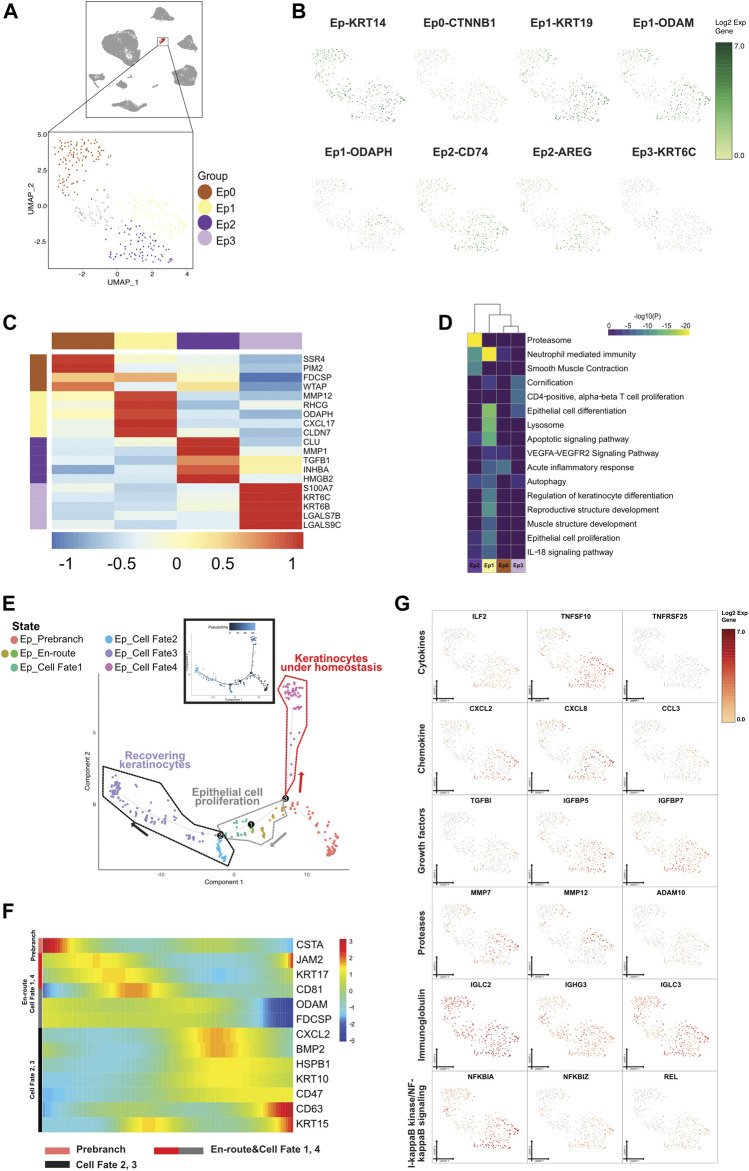
A single-cell atlas for characterizing epithelial cells. **(A)** The UMAP plot presents the distribution of the epithelial cell cluster (red, *n* = 332) in the atlas (top), and the enlarged UMAP plot (bottom) shows the four subclusters of epithelial cell populations (color coded). **(B)** The feature plots (UMAP) display the main signatures for the epithelial cells. ODAM is the Malassez marker as previously studied. **(C)** The heatmap showing the top 5 key differentially expressed genes [by average log (fold change)] from the epithelial cell subcluster. **(D)** The heatmap showing the corresponding enriched pathways of the four epithelial subtypes [by average log (fold change)]. **(E)** The differentiation pseudotime trajectory analysis. Prebranch cells (soft red, E87D72) are classified into the different numbered cell fates according to the different branches. The cells in the Ed_en routes (moderate yellow, BD9C33 and moderate cyan-lime green, 56BE97) illustrate the condition cells were in before branching. Epithelial cells in cell fate 1 (moderate lime green, 53B74C), cell fate 2 (soft magenta, E96BD2), cell fate 3 (soft blue, 51B4E6), and cell fate 4 (purple color, A18BF8) are displayed. Each dot represents a single cell, and is colored according to its cluster label. **(F)** The DEGs in rows along the pseudotime are hierarchically clustered into three subclusters, representing each epithelial subpopulation’s cell fate. The clustering of genes according to direction and timing reveals that epithelial cells are remodeled in different trends in response to inflammation. **(G)** Relative response- or signaling pathway-associated changes in gene expression are visualized in a UMAP plot.

We classified Ep into 4 cell subclusters according to marker genes (Figures 7A–D). Ep0 were annotated as a group of proliferating epithelial, and implicated phagocytosis and the recognition process. Ep1 were related to odontogenesis of dentin-containing tooth, indicating that this cell subcluster might be originated from ERM. Ep2, with expression of myeloid (CD74), epithelial cell markers, growth factors, and cell cycling markers (HMGB2 and CDK1), might be differentiated from myeloid cells in an inflammatory environment. Ep3 were associated with proliferating T cells and involved in keratinization.

#### 3.5.2 Lineage Bifurcation in the Epithelial Differentiation Trajectory

Eps underwent an abnormal differentiation program from immune tolerogenic mesenchymal cells and proliferative layer cells to corneocytes, with TFs differentially expressed between the cell states (Figures 7E–G and Supplementary Figure S7B). Cells characterized with the precursor envelopment of cornified cells (CSTA) in the prebranch exerted an immune response by lymphocyte activation. In view of the enhancement of MMP12 and the presence of TGFβ3, cells in cell fate 1 were enriched in epithelial cell proliferation and were in the process of epidermal development after being triggered by an attack of inflammatory factors (IL2RG and NFκBIL1 existed in Ep_enroute). Cells in cell fate 2 highly expressed antimicrobial genes (CXCL1) and executed apoptosis (RACK1, UBB, TPT1, S100A9, MIF, and ATF3). Along with the emergence of IGFBP2, IGFBP5, IGFBP7, IMMP1L, MMP13, and MMP7 and decrease of CXCL2, ILF2, IL20RB, TNFRSF1A, TNFSF10, TNFAIP3, and TNFRSF12A, cells in cell fate 3 were annotated as recovering keratinocytes with a high level of epithelial cell markers (KRT14, KRT19, and KRT5). As mentioned above, proliferating keratinocytes from prebranch to cell fates 1, 2, and 3 were a convincing aberrant signal for cell morphogenesis of the epithelium. Following the high expression of mitochondrially encoded gene and low regulator lymphocyte homeostasis (TNFRSF25), cells in cell fate 4 were correlated with keratinization, which indicated that the cells referred to a population present in the lesions under homeostasis.

### 3.6 Cellular Communications in CAP

**FIGURE 8:**
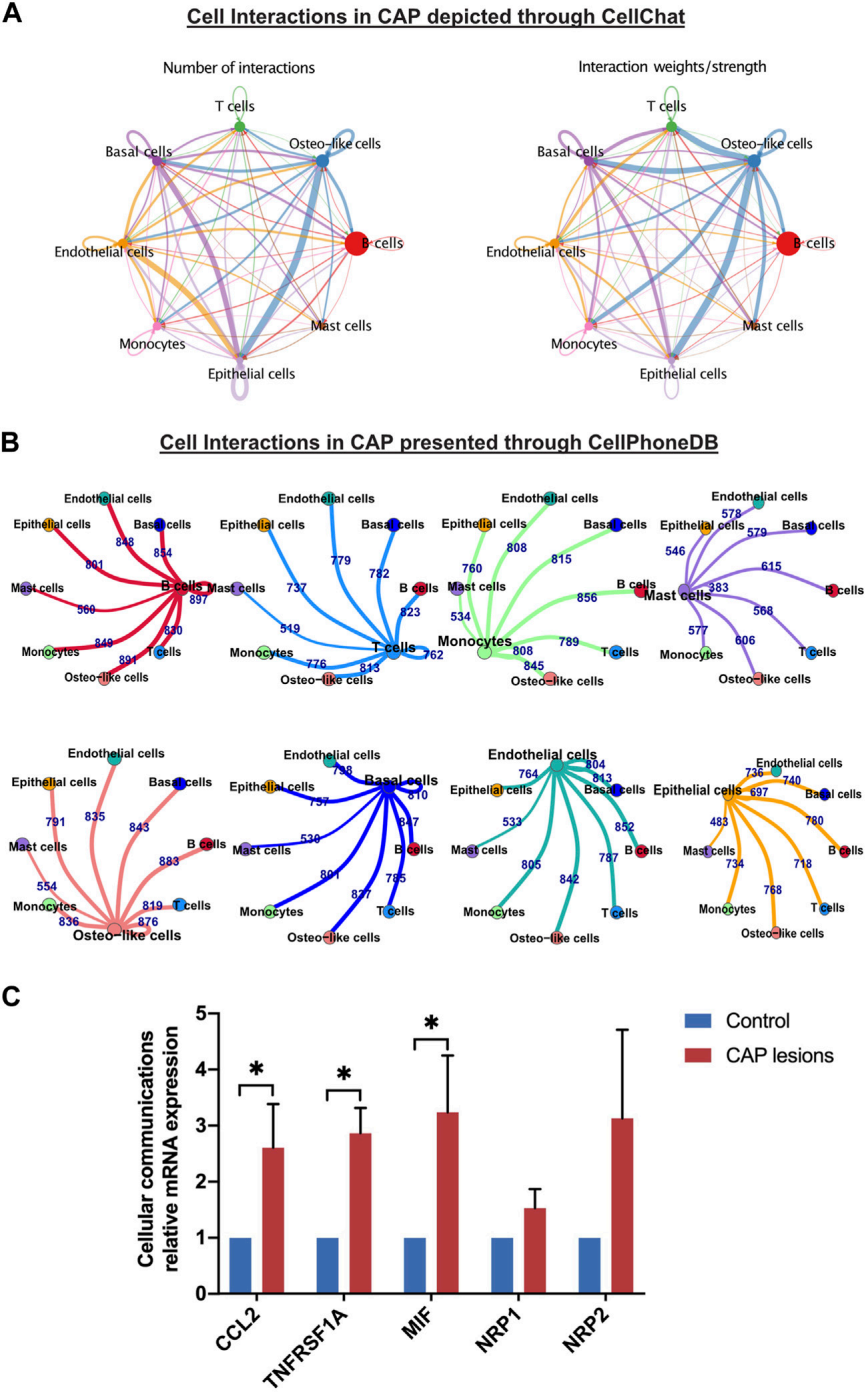
Differentially expressed genes and cell–cell interactions in CAP. Cell–cell interactions in CAP are depicted through CellChat **(A)** and presented through CellPhoneDB **(B)**. Relative mRNA expressions of some key predicated genes in cell–cell interactions between nonimmune cells and the other types of cells were evaluated by qRT-PCR **(C)**. The validation was repeated from three samples of periapical tissue of CAP. *p < 0.05 compared to the control group.

Crosstalk between neighboring cells in CAP triggers multiple processes, including signal transductions and genomic presentations, and generates biological functions. Our single-cell transcriptomic analysis of periapical samples of CAP presented eight cell types that communicate *via* ligand–receptor interactions. The CCIs between immune cells and nonimmune cells showed tight relationships by CellChat and CellPhone DB (Figures 8A,B), which modulated the development of CAP. We unveiled interaction between the cell types based on the increased expression of these chemokines, cytokines, growth factors, and their receptors in CAP (Supplementary Figure S8). In the four nonimmune cells, osteo-like cells had the most chemokines. CXCL12 in osteo-like cells interacted with the receptor CXCR4 in all subclusters. The three nonimmune cells, osteo-like, basal/stromal, and endothelial cells, showed the CCL2–ACKR1 interactions. Two ligand–receptor pairs, macrophage migration inhibitory factor (MIF)–TNFRSF14 and TNFRSF1A–GRN, were enriched in the most nonimmune cells, suggesting that MIF and TNFRSF1A played a role in the interaction with other cell clusters in the development of CAP. The ligand EGFR secreted by epithelial and osteo-like cells generally interacted with several receptors such as TGFβ1, MIF, AREG, GRN, COPA, and HBEGF in other cell types. Furthermore, ligand NRP1 and NRP2 secreted by the four nonimmune cells interacted with the receptor VEGFA and PGF in the other cell types. The high expression of the ligands CCL2, TNFRSF1A, MIF, NRP1, and NRP2 in CCIs were detected by qRT-PCR (Figure 8C).

### 3.7 Aberrant Transcription Factors Profiled in Nonimmune Cells of CAP at Single-Cell Resolution

Nonimmune cells in the periapical lesions of CAP were functionally specialized by detecting distinct gene expression in the differentiated cell subclusters described above. Perturbations in TFs caused the alterations of cell biological function, ranging from external defense stimuli to impairment of barrier function in an inflammatory environment. TFs in specific cell types may regulate cell type-specific functions for their respective reaction mechanisms. We utilized TRRUST to predict 20 key TFs modulating nonimmune cell subclusters (Supplementary Figure S9). For example, in subclusters of osteo-like, basal/stromal, and endothelial cells, regulatory factor X (RFX5, RFXAP, and RFXANK) can mediate their immunodeficiency function by binding domains of RFX- and NFX-. Considering the wide existence of nuclear factor-kappaB (NF-κB) and proto-oncogene (RELA), nonimmune cells express immunological responses to confront infections. Zinc finger TF (SP1) in nonimmune cells protected tissue from being harassed by inflammatory factors. These TFs indicated that tissue remodeling by promoting cellular development was involved in the enormous relevant signaling pathways.

**FIGURE 9:**
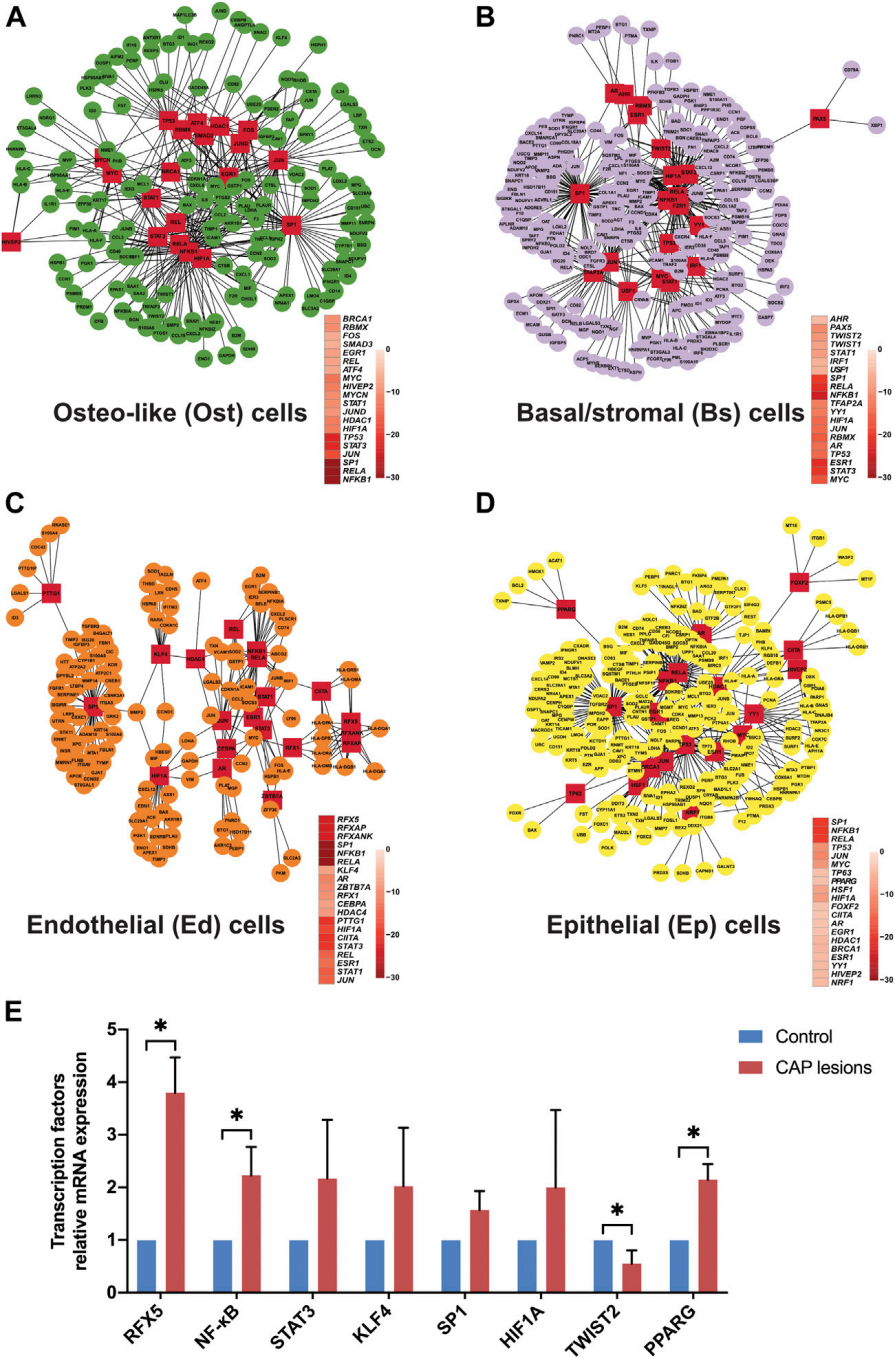
The forecasted transcriptional factors of nonimmune cells in CAP lesions. Twenty key transcription factors and relative target genes are predicted by dynamic variations in gene expression in osteo-like cells **(A)**, basal/stromal cells **(B)**, endothelial cells **(C)**, and epithelial cells **(D)** from common CAP to typical periapical granuloma. Cofunctional networks were associated with the inflammatory response and nonimmune cell development. The red square nodes represent TFs, and the round nodes represent target genes. The correlated TFs in cell fates are shown. Relative mRNA expressions of some forecasted TFs in periapical tissue of CAP were further compared with the normal periapical tissue by qRT-PCR **(E)**. *p < 0.05 compared to the control group.

To explore an immune checkpoint for further CAP therapy, we forecasted 20 potential TFs to distinguish the cell fates of nonimmune cells through a multifaceted mechanism (Figures 9A–D and Supplementary Figure S10). Under inflammatory stimuli, intermediate progenitor cells of nonimmune cells that respond to lineage-specific inducers can be differentiated into mutually exclusive lineages. In the oxygen-deprived environment like CAP, hypoxia-inducible factor 1 subunit alpha (HIF1A) in nonimmune cells is a critical TF that reduces the synthesis of inflammatory cytokines and thus minimizes local damnification by stimulating its target genes (Wang et al., 2014). From the data, the shared TFs in nonimmune cells were related to tissue repair, including angiogenesis, osteogenesis, and adipogenesis. In osteo-like cells, the TF STAT3 might be activated by inflammatory cytokines (TNFRSF21, TNFAIP3, TNFRSF12A, TNFRSF11B, and IL6 in ost_cell fate 1) and growth factors (BMP2 in ost_prebranch and ost_cell fate 1) through kinase-mediated tyrosine phosphorylation and dimerization. The tumor suppressor TP53 might interact with BAX (only in ost_cell fate 1), leading to cellular apoptosis. In parallel, the twist family BHLH TFs (TWIST1 and TWIST2) repressed proinflammatory cytokines and carried out intercellular communication between Ost cells and Bs cells. TWIST2 can also inhibit early or ectopic differentiation of osteoblasts in the process of osteogenesis, which indicated that fibrosis in local lesions of CAP indirectly hinders bone regeneration. In endothelial cells, Krüppel-like factor 4 (KLF4) plays a dual role as a transactivator or as a transrepressor in modulating the expression of its distinct target genes. The homeostasis of epithelial cells is predisposed to be regulated by KLF4, resulting in cell proliferation, metastasis, angiogenesis, or inhibition of suppressive effects by apoptosis, differentiation, and cell cycle arrest. Peroxisome proliferator-activated receptor gamma (PPARG) participates in sustaining tissue homeostasis primarily by attenuating NFκB-mediated proinflammatory responses. It is therefore plausible that the modulation of TFs in the immune response causes developmental dysfunctions in local lesions and in turn regulates the tissue regeneration of nonimmune cells.

In view of the above prediction of TFs in this bioinformatic analysis, we further identified and compared some key TFs in normal periapical tissues and CAP lesions by qRT-PCR. RFX5, NF-κB, STAT3, KLF4, SP1, HIF1A, and PPARG showed high expression while TWIST2 displayed low expression in three inflammatory periapical tissue compared to the control (Figure 9E), which was in accord with the prediction in single-cell resolution.

## 4 Discussion

As artificial platforms unravel the fate and function of progenitor cells and effector cells in physiological and pathological processes (Reid and Wernisch, 2016), single-cell technologies were applied to parse cellular genomic signals and aberrations in CAP. Our high-throughput screening data were consistent with the holistic cell types known to be present in CAP through traditional pathological sections (Bănică et al., 2018). Though human teeth had been mapped the transcriptional landscape of the various cell populations at single-cell resolution (Krivanek et al., 2020; Pagella et al., 2021), to our knowledge, cellular heterogeneity in lesions of human CAP has not been studied hitherto. The hyperplastic inflammatory periapical tissue, by the infection the teeth set up, occurs in the alveolar bone, but we difficultly collect enough soft tissue to be control in healthy alveolar bone. Severe periapical tissue destruction, inflammatory granulation tissue formation, and alveolar bone resorption are the hallmarks of CAP. The pathological change of diseases usually develops through the course from small to large. Likewise, CAP develops from the small to large hyperplastic inflammatory periapical tissue. In the three samples, the size of Sample C was significantly bigger than Samples A and B. As a result, we primarily considered differentiation trajectory from common periapical inflammatory to granuloma state according to the size of hyperplastic inflammatory periapical tissue. We profiled the extensive picture of nonimmune cell in CAP, presented the complexity of participating cells, and charted the differences in frequencies and molecular states. The remarkable alterations of nonimmune cell type-specific gene expression signatures have been reckoned by cellular perturbations driven by inflammatory factors.

Single cells, the building blocks of tissue systems that organize organs in the body, furnish living organisms with phenotypes, behaviors, and functions (Hedlund and Deng, 2018). Here, we obtained common chronically inflamed periapical tissues and typical periapical granuloma from three patients and evaluated the cells belonging to the same lineage within clusters *via* unsupervised clustering analysis. Notably, the overall transcriptional programs of the effector cells were similar enough for an unbiased algorithm despite the discrepancies among the inflammatory periapical tissues. Immune cells coordinate mature conventional tissue cells undergoing early inflammatory responses, and concomitantly, inflammatory cytokines drive the transformation of intermediate native progenitor cells with multipotency capacity.

One further matter of interest in the responsive cellular populations in inflammatory periapical tissue is the extent to which nonimmune cells interact with immune cells. In the inflammatory tissue of CAP, lymphocytes and myeloid cells intermingled in nonimmune cells were epigenetically wired by local microenvironmental cues to regulate the innate immune system and conduct inflammatory stimuli. Pseudotime trajectory analysis revealed that asynchronous differentiation of nonimmune cells might be due to the drive of the inflammatory and growth factors. By analyzing the distinct gene expression of nonimmune cells from two common CAPs and a typical periapical granuloma, we speculated that foreign bodies might initially stimulate mature B lymphocytes intermingled in nonimmune cells to produce immunoglobulin-mediated immunity. An inextricable link between the alterative inflammatory cytokines and the intermingled immune cells in nonimmune cells resulted in outstanding consequences regarding damaged periapical tissues. The extensive chemokines and growth factors in the inflammatory nonimmune cells in our data increased the possibility that nonimmune cells were activated by their emergence, but the intervention of negative regulation of the immunological system could not be ignored.

Nonimmune cells are capable of plasticity during inflammation, reinforcing the dogma of negative modulation of the immune system by presenting hyperplastic lesions due to the degradation of collagen and mineral and cellular imbalance in aging and inflammation. The nonimmune cells, including the osteo-like, basal/stromal, and epithelial cells, are considered resident sentinel cells in local lesions of CAP except for endothelial cells. The confluence of osteoclastic cell differentiation and osteoblastic cell redifferentiation under inflammatory stimulation results in bone absorption in CAP. Additionally, keratinization and inflammatory-associated fibroblast and myofibroblast formation indicate intercellular communication, which is important for tissue remodeling in response to perturbations in periapical tissue homeostasis. During the early inflammatory response, the presence of various immunoglobulins partially limits impairment of nonimmune cell, but culminates in uncontrolled immune responses against the sentinel cells mentioned above. We found a unique impactful role of endothelial cells by presenting lymphangiogenesis and angiogenesis in the progression of CAP. Furthermore, we unveiled the enormous heterogeneity in cellular phenotypic characterizations and function of the nonimmune cells through the predicted putative biological process regulated by the relevant TFs.

Significantly, the mirrored data profiles between single-cell sequence analysis and immunohistochemistry staining displayed the presence of the eight types of cells in CAP including nonimmune cells and immune cells. These observations shed light on periapical lesions and identify targets to interpret the pathological mechanisms from common CAP to typical periapical granuloma. Our study underscored the heterogeneities of effector cells and the peculiarity of immune mechanism in periapical lesion. The long-term exposure of nonimmune cells to proinflammatory host mediators, chemokines (interleukins and tumor necrosis factors), and bacterial production induces cellular epigenetic alterations, and results in cell apoptosis and tissue damage. In general, dramatic genomic changes in CAP may reflect how the microenvironment constrains cellular characterization. Further research should focus on defining the relevant countermeasures by immunological interventions to explore an immune checkpoint and ameliorate the lesion in CAP.

The cellular communication analysis using the scRNA-seq data uncovered the possible roles of ligand–receptor-mediated CCIs. Differential regulation of ligand–receptor interactions between cell populations in CAP was identified by comparing the number of single-cell/single-cell ligand–receptor interactions and interaction weights/strength, implying a potential therapeutic target for CAP. For putative ligand–receptor pairing validation, qRT-PCR was conducted using three periapical tissues of CAP. In our data, MIF–TNFRSF14 is the most enriched cytokine ligand–receptor pair in all nonimmune cells during the development of CAP (Supplementary Figure S10). MIF was defined as an important chemokine-like function chemokine with an essential role in monocyte recruitment and arrest, and considered to be critical in atherogenesis and inflammatory diseases (Tillmann et al., 2013). The receptors CXCL2, CXCL4, and CD74 have been identified to interact with MIF and mediate MIF-triggered arrest functions (Bernhagen et al., 2007; Tillmann et al., 2013; Wu et al., 2019). Notably, we first found MIF in nonimmune cells bound to the receptor TNFRSF14 in other types of cells. TNFRSF14, known as herpesvirus entry mediator (HVEM), encodes members of the tumor necrosis factor (TNF) receptor superfamily and serves as a molecular switch by interacting with different ligands to regulate a series of immune responses (Yu-Di and Ming-Yue, 2018; Wang L. et al., 2021). TNFRSF14 mediates apoptosis and prevents tumor cells from immune escape. Therefore, we speculated that MIF–TNFRSF14 interaction between nonimmune cells and other types of cells was conducive to the recovery of CAP. The exact mechanism of the MIF–TNFRSF14 pair in CAP needs to further be studied in the future.

### 4.1 Limitations of the Study

Current single-cell transcriptional technologies unveiled multidimensional cellular data in CAP and provided insight into the cellular communities and pathogenic signaling pathways involved. It is the first time that we employed single-cell transcriptional technologies to analyze the cell-type composition and transcriptome data in lesions of human chronic apical periodontitis. However, due to the limitation of number of the specimens, the acquired biological information still needs to be validated in the future.

## Data Availability

The datasets presented in this study can be found in online repositories. The names of the repository/repositories and accession number(s) can be found below: The accession number for all sequencing data reported in this paper is GEO: GSE181688. Further inquiries can be directed to tongzhch@mail.sysu.edu.cn.
