# Supplementary figures and images for: Single-Cell Sequencing Unveils the Heterogeneity of Nonimmune Cells in Chronic Apical Periodontitis
Source: Front Cell Dev Biol. 2022 Feb 10;9:820274. doi: 10.3389/fcell.2021.820274 (PMC8883837; doi:10.3389/fcell.2021.820274)

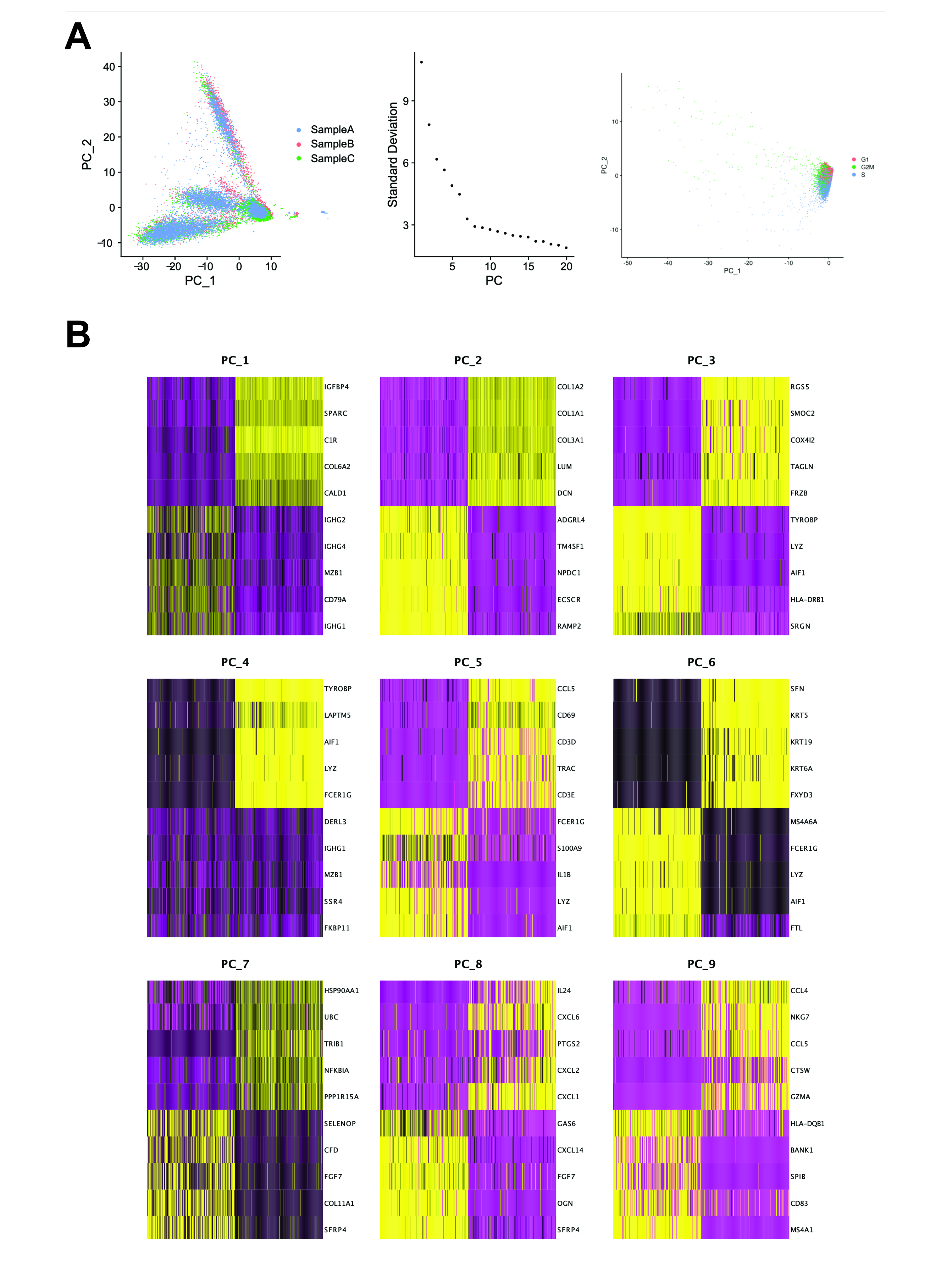

Supplement: Supplementary file 1 [file Image3.JPEG]

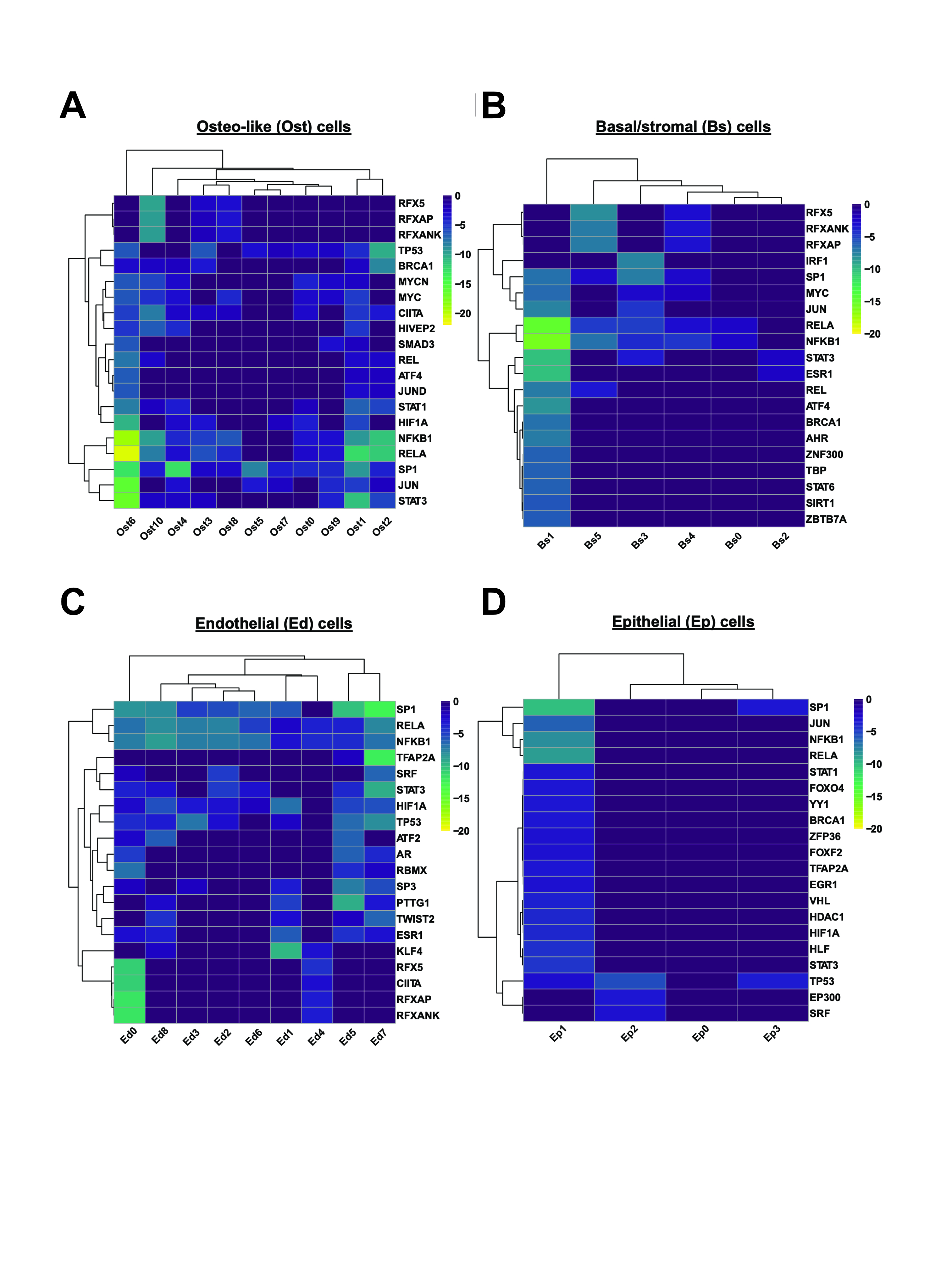

Supplement: Supplementary file 2 [file Image9.JPEG]

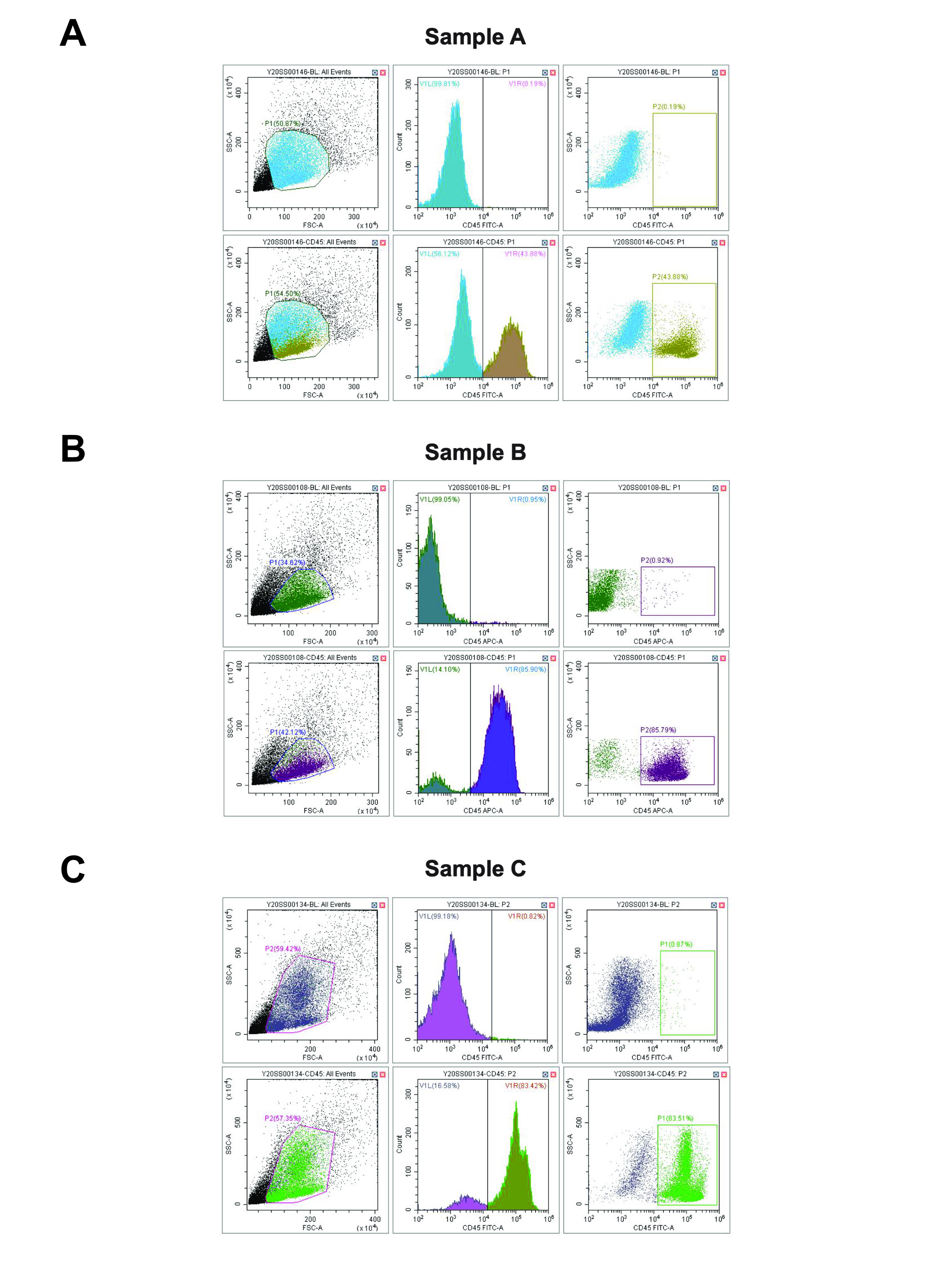

Supplement: Supplementary file 3 [file Image1.JPEG]

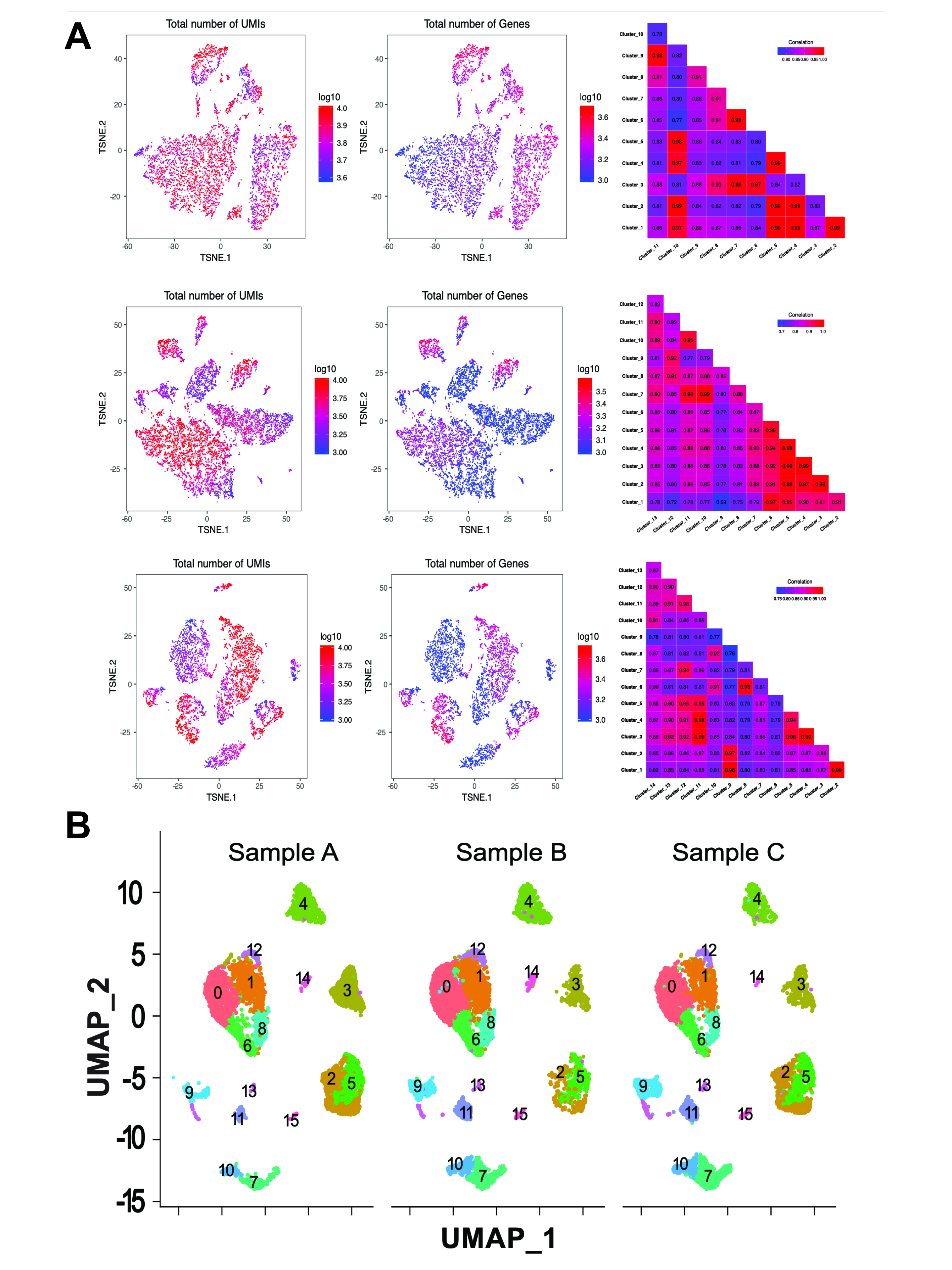

Supplement: Supplementary file 4 [file Image4.JPEG]

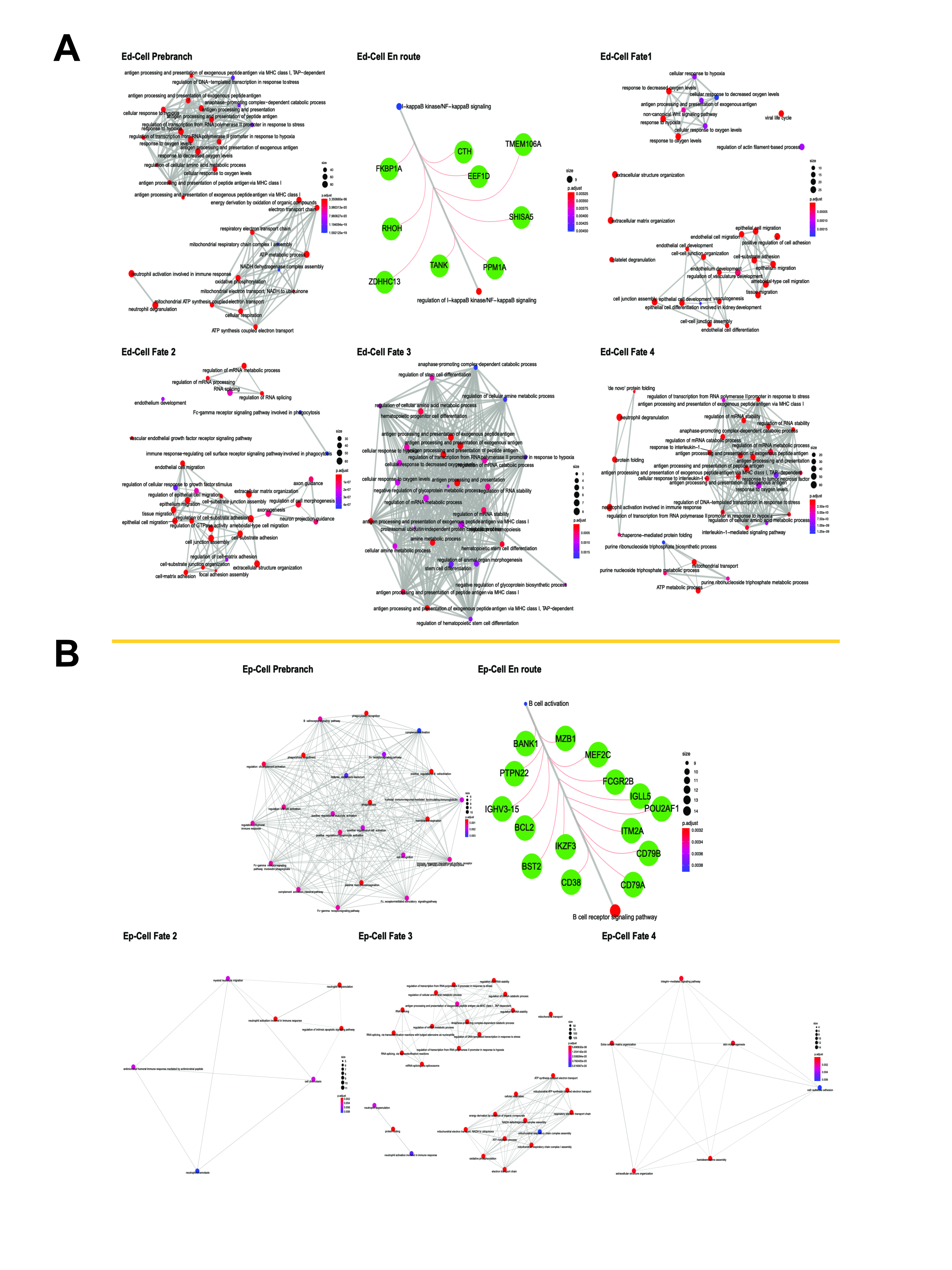

Supplement: Supplementary file 5 [file Image7.JPEG]

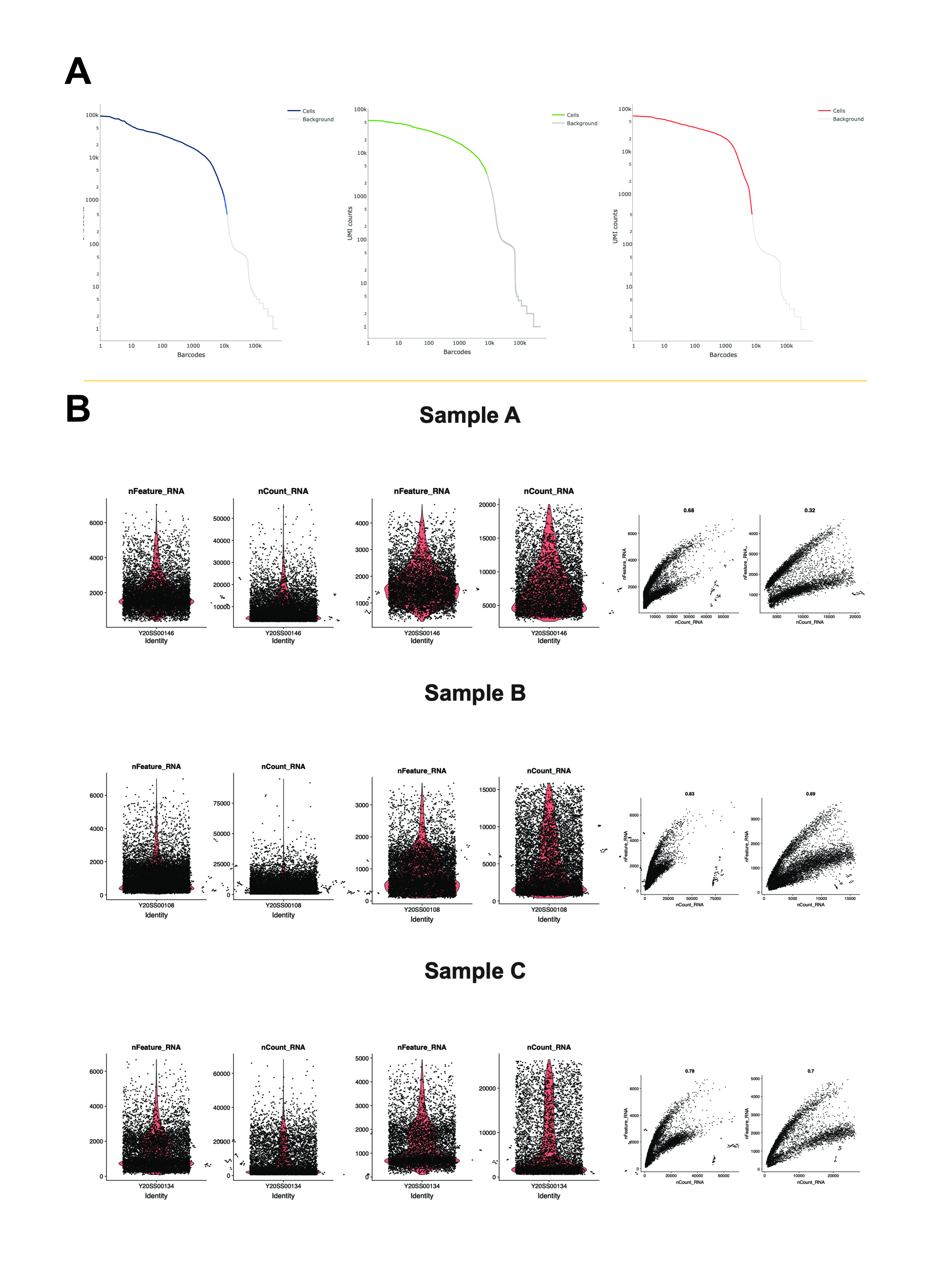

Supplement: Supplementary file 6 [file Image2.JPEG]

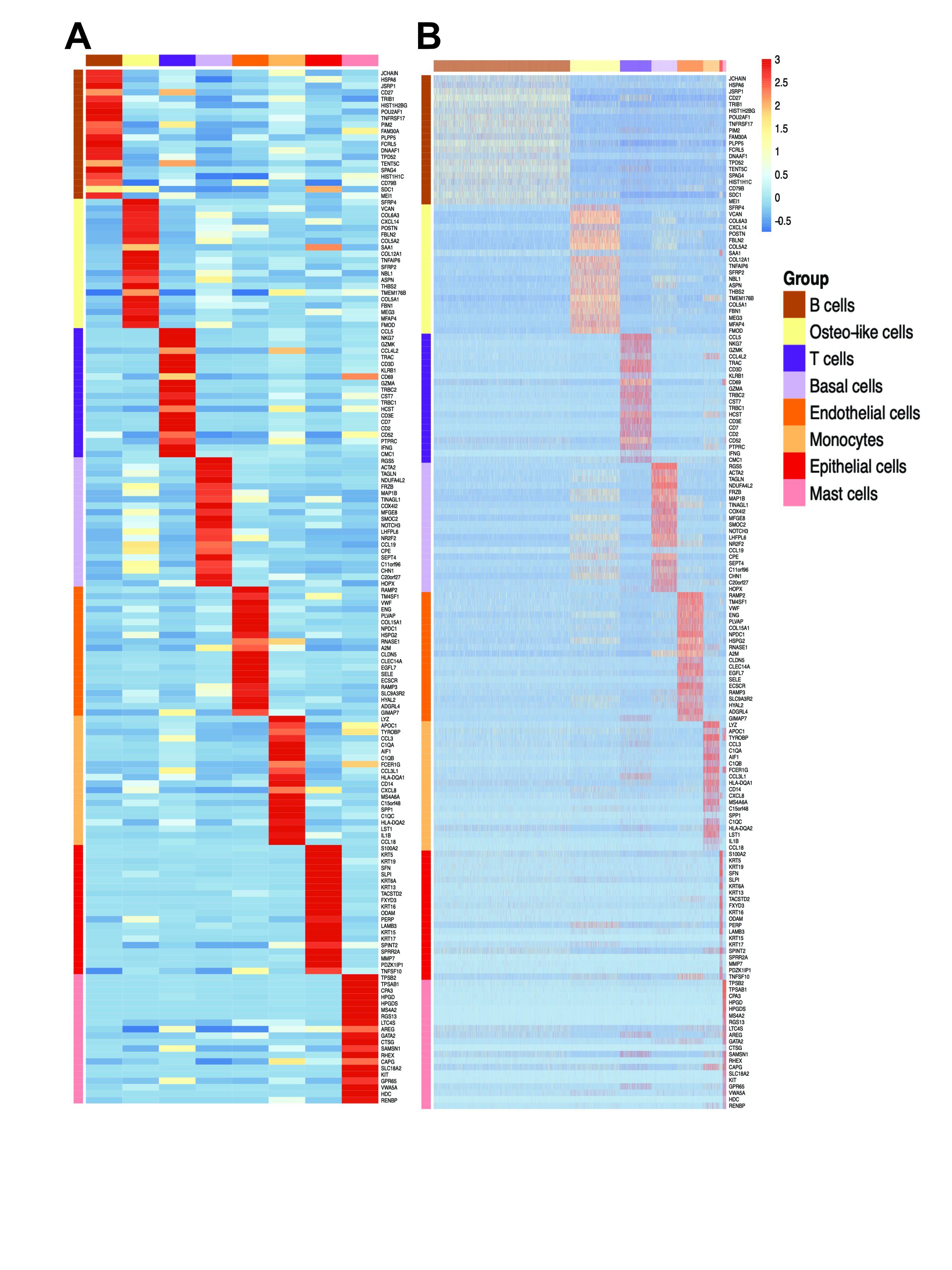

Supplement: Supplementary file 7 [file Image5.JPEG]

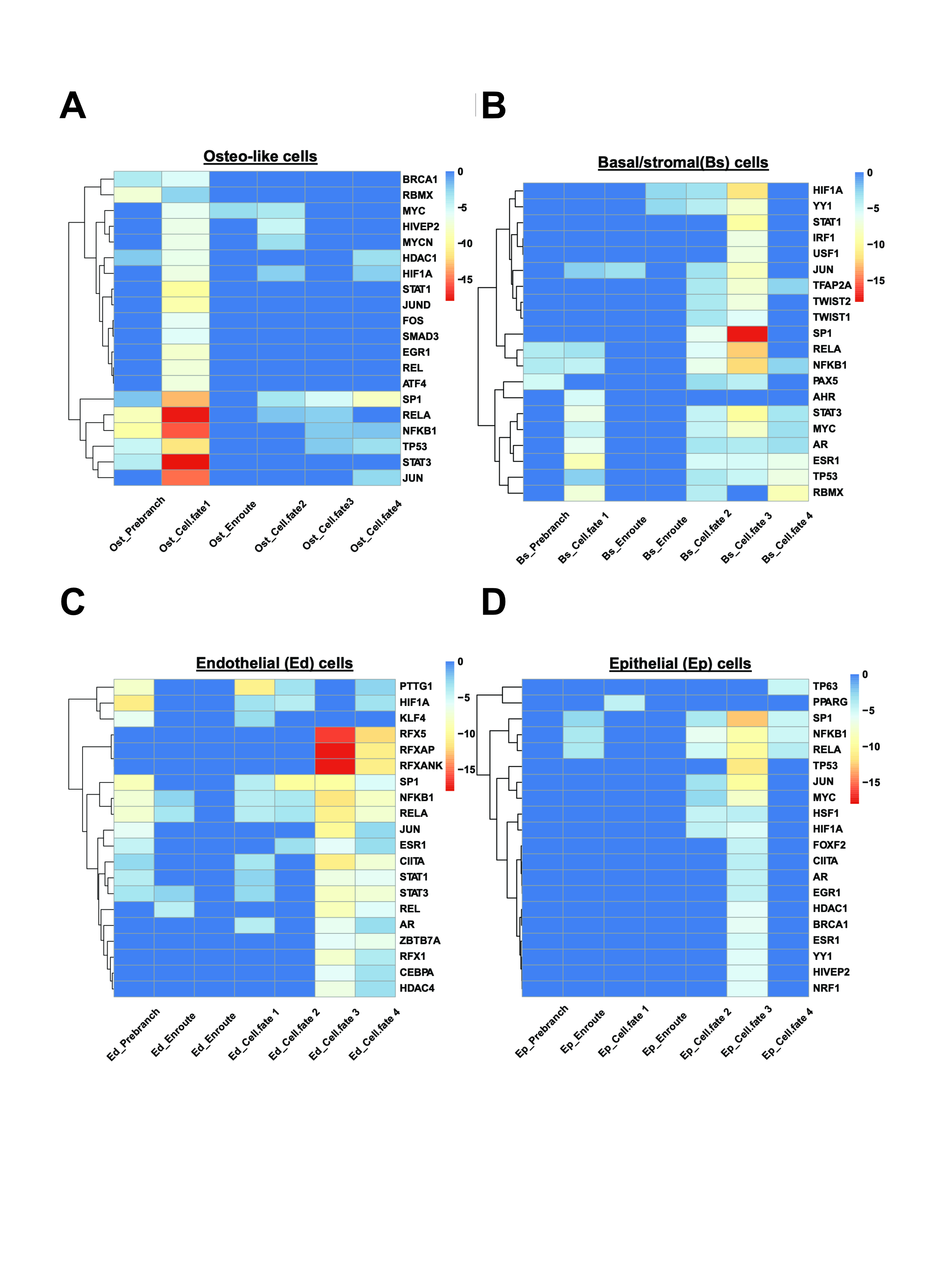

Supplement: Supplementary file 8 [file Image10.JPEG]

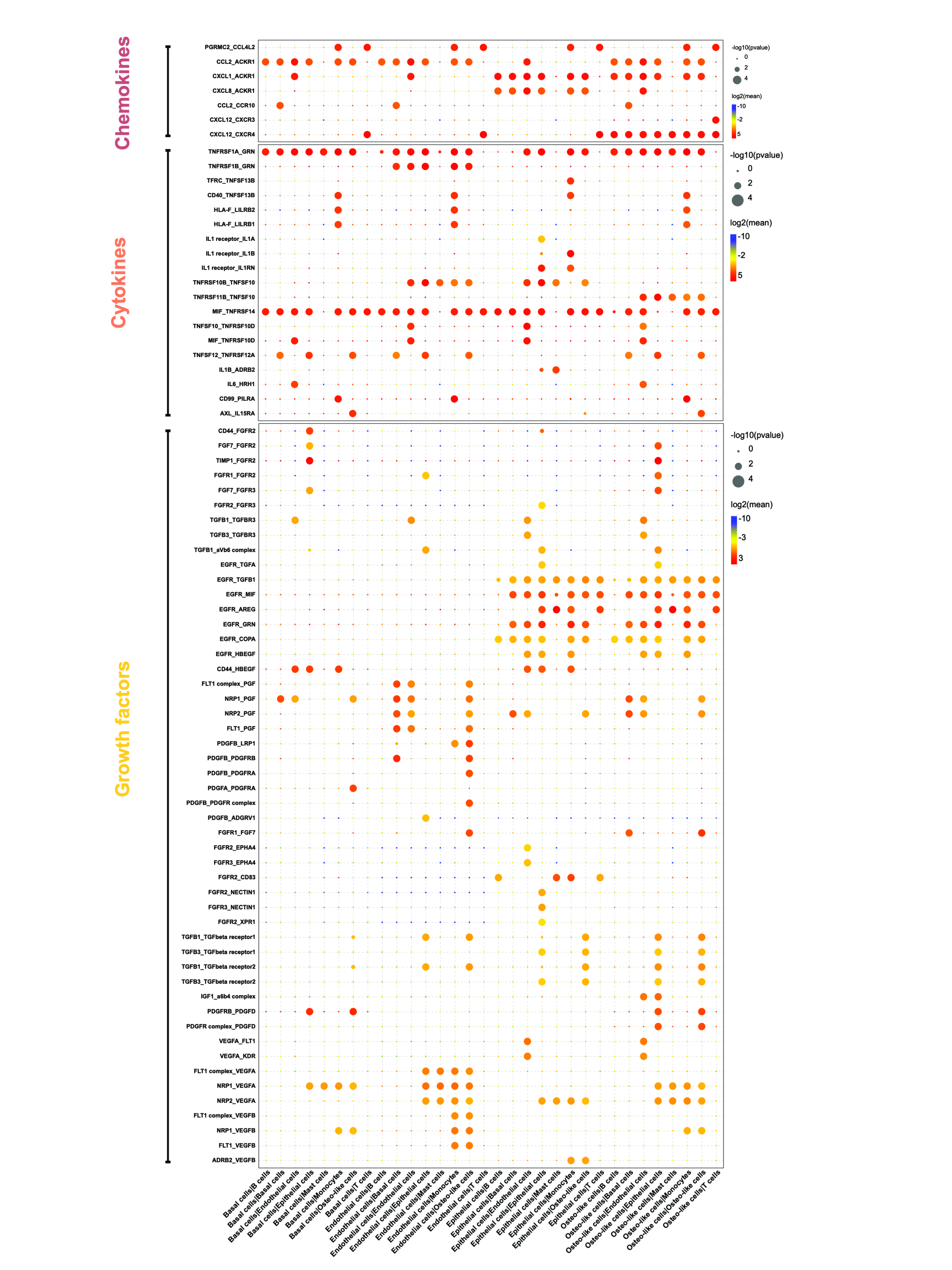

Supplement: Supplementary file 9 [file Image8.JPEG]

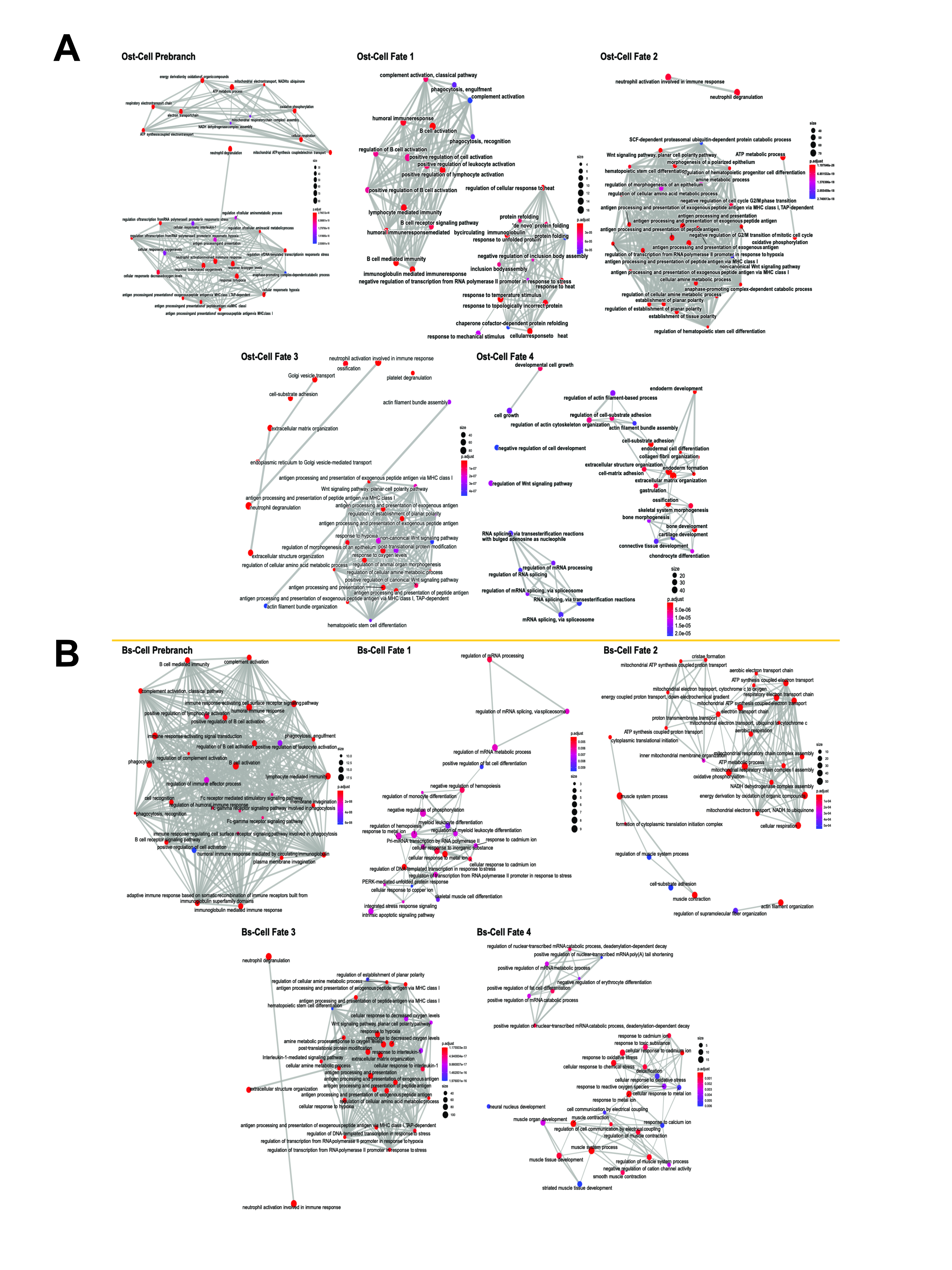

Supplement: Supplementary file 11 [file Image6.JPEG]
