# Supplementary material for: Single-Cell Sequencing Unveils the Heterogeneity of Nonimmune Cells in Chronic Apical Periodontitis
Source: Front Cell Dev Biol. 2022 Feb 10;9:820274. doi: 10.3389/fcell.2021.820274 (PMC8883837; doi:10.3389/fcell.2021.820274)
Supplement: Supplementary file 10 [file DataSheet1.DOCX]

**Supplementary Figure Legends**

**Sup Fig 1. Cell sorting for single-cell sequencing**

Representative flow cytometry analysis showing cell viabilities and CD45-positive cell populations in Samples A, B and C.

**Sup Fig 2. Unbiased clustering analysis of 3 specimens**

a. Cumulative fraction (y-axis) of the UMI counts per cell (x-axis) for sample A (dark blue solid lines), sample B (green solid lines) and sample C (red solid lines).

b. Quality control of scRNA-seq. The cells with poor quality were filtered out, and the positive associations between detected gene counts and sequencing depth were assessed.

**Sup Fig 3. PCA across each component**

a. Variation in gene expression driven by cell cycle stages and mitochondrial gene expression were removed to reduce the variance introduced by unwanted sources.

b. The top 9 components from the PCA procedure are shown and exhibit the correlated genes in each component. The colors ranging from purple to golden yellow represent the expression levels of correlated genes from low to high.

**Sup Fig 4. Integration and comparison of human CAP**

a. T-SNE depicting unbiased clustering of inflammatory cell data sets separated from three sequentially described samples. A T-SNE plot was visualized to divide the cells into different clusters according to the actual cell type. The right side illustrates the correlation between each cluster in the same sample. The higher the correlation was, the closer the expression trend of cells was in the cluster.

b. Each dot represents a single cell colored according to its cluster label. Its diversity was determined by nonlinear dimensionality reduction using the U-MAP algorithm.

**Sup Fig 5. Characterization of single-cell RNA sequencing for 8 main clusters and screening of marker genes**

The heatmap shows the top 20 key differentially expressed genes (by average log[fold change] between 8 clusters we identified. Each row represents one gene, and each column represents a single cluster.

**Sup Fig 6. Differentially regulated GO terms for each cell fate (state) in pseudotime analysis**

a. A functional enrichment analysis based on the gene ontology (GO) database was demonstrated based on differentially expressed genes in osteo-like (Ost) cells.

b. According to the differentially expressed genes in the basal/stromal (Bs) cells, a functional enrichment analysis based on the gene ontology (GO) database was performed.

The similarity index was calculated by the Pearson correlation coefﬁcient.

**Sup Fig 7. Differential expression analysis of GO terms for each cell fate (state) in pseudotime analysis**

a. Functional annotations based on differentially expressed genes of endothelial (Ed) cells are shown in the gene ontology (GO) database.

b. Functional enrichment analysis based on the gene ontology (GO) database was employed by differential expression of epithelial (Ep) cells.

**Sup Fig 8. The interaction between the cell types in CAP**

The interaction between the cell types based on the increased expression of these chemokines, cytokines, growth factors and their receptors in CAP.

**Sup Fig 9. Prediction of key transcription factors (TFs) for the nonimmune cell subtypes**

a. A heatmap shows the prediction of 20 key transcription factors (TFs) for osteo-like cells (Ost) in each subcluster.

b. The 20 top-ranked transcription factors (TFs) are shown in a heatmap predicted to regulate the basal/stromal (Bs) cell subtypes.

c. A heatmap visualization of color-coded endothelial cells (Ed) per subcluster (stacked rows) for 20 significantly predicted transcription factors (columns).

d. A heatmap showing the 20 key predicted transcription factors in the epithelial cell (Ep) subclusters.

**Sup Fig 10. Pseudotime analysis traces the nonimmune cells from the common periapical inflammatory to granuloma states by predicted key transcription factors (TFs)**

a. The heatmap showing the prediction of 20 key transcription factors (TFs) regulated by osteo-like cells (Ost) in each cell fate.

b. The 20 top-ranked transcription factors (TFs) shown in a heatmap are predicted to regulate basal/stromal (Bs) cells at various stages of differentiation.

c. The heatmap visualization of the 20 key predicted transcription factors for regulating the coding of endothelial cells (Ed) per cell fate.

d. The heatmap showing the 20 key predicted transcription factors for the cell fate commitment of epithelial cells (Ep) under the inflammatory response.
